# MACS: Rapid Aqueous Clearing System for 3D Mapping of Intact Organs

**DOI:** 10.1002/advs.201903185

**Published:** 2020-02-25

**Authors:** Jingtan Zhu, Tingting Yu, Yusha Li, Jianyi Xu, Yisong Qi, Yingtao Yao, Yilin Ma, Peng Wan, Zhilong Chen, Xiangning Li, Hui Gong, Qingming Luo, Dan Zhu

**Affiliations:** ^1^ Britton Chance Center for Biomedical Photonics Wuhan National Laboratory for Optoelectronics Huazhong University of Science and Technology Wuhan 430074 China; ^2^ MoE Key Laboratory for Biomedical Photonics Huazhong University of Science and Technology Wuhan 430074 China

**Keywords:** 3D mapping, aqueous clearing systems, fluorescence neuroimaging, lipophilic dyes, tissue clearing, vasculature, visualization

## Abstract

Tissue optical clearing techniques have provided important tools for large‐volume imaging. Aqueous‐based clearing methods are known for good fluorescence preservation and scalable size maintenance, but are limited by long incubation time, insufficient clearing performance, or requirements for specialized devices. Additionally, few clearing methods are compatible with widely used lipophilic dyes while maintaining high clearing performance. Here, to address these issues, m‐xylylenediamine (MXDA) is firstly introduced into tissue clearing and used to develop a rapid, highly efficient aqueous clearing method with robust lipophilic dyes compatibility, termed MXDA‐based Aqueous Clearing System (MACS). MACS can render whole adult brains highly transparent within 2.5 days and is also applicable for other intact organs. Meanwhile, MACS possesses ideal compatibility with multiple probes, especially for lipophilic dyes. MACS achieves 3D imaging of the intact neural structures labeled by various techniques. Combining MACS with DiI labeling, MACS allows reconstruction of the detailed vascular structures of various organs and generates 3D pathology of glomeruli tufts in healthy and diabetic kidneys. Therefore, MACS provides a useful method for 3D mapping of intact tissues and is expected to facilitate morphological, physiological, and pathological studies of various organs.

## Graphical abstract

Here, a rapid, highly efficient aqueous clearing method is described, termed the m‐xylylenediamine (MXDA)‐based Aqueous Clearing System (MACS). MACS enables ultrafast clarification on intact organs with high transparency and simultaneously possesses excellent compatibility with lipophilic dyes. MACS may provide a cutting‐edge method for rapid clearing and volumetric imaging for multiple biological structures and promote comprehensive morphological and pathological studies of intact organs or bodies.

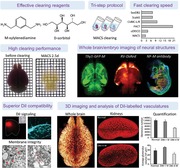

## 1 Introduction

3D imaging of tissue structures at high resolution plays an indispensable role in life science. The development of diverse fluorescent labeling methods and optical imaging techniques provides essential tools for 3D imaging of large‐volume tissues.^[^ 1, 2, 3, 4, 5 ^]^ However, the imaging depth is rather limited due to the opaqueness of the tissue.^[^ 6 ^]^ Automated serial‐sectioning and imaging techniques have been developed to address this issue, allowing the acquisition of high‐resolution images throughout the brain.^[^ 7, 8, 9, 10 ^]^

As a distinct solution, tissue optical clearing technique has been proposed for imaging deeper without cutting.^[^ 11, 12, 13, 14 ^]^ In the past decade, a variety of optical clearing methods have been proposed, providing powerful tools for visualizing large volume tissues. They are principally divided into two categories, namely solvent‐based methods and aqueous‐based methods. The solvent‐based methods can achieve high level of tissue transparency and substantial tissue shrinkage within several days, but are faced with the problem of quenching endogenous fluorescence signals, such as 3D imaging of solvent‐cleared organs (3DISCO).^[^ 15, 16 ^]^ In recent years, some of the latest solvent‐based methods, such as iDISCO (immunolabeling‐enabled DISCO),^[^ 17 ^]^ uDISCO (ultimate DISCO),^[^ 18 ^]^ PEGASOS (polyethylene glycol (PEG)‐associated solvent system),^[^ 19 ^]^ ethanol‐ECi,^[^ 20 ^]^ FDISCO (DISCO with superior fluorescence‐preserving capability),^[^ 21 ^]^ vDISCO (nanobody(V_H_H)‐boosted DISCO),^[^ 22 ^]^ and sDISCO (stabilized DISCO),^[^ 23 ^]^ can address this issue to some extent. The aqueous‐based methods, as another category, are known for good fluorescence preservation and scalable maintenance of tissue size. However, for organ‐scale tissues, they suffer from either long clearing time, or insufficient clearing performance, or complexity of handling. To address these issues, many groups have made great efforts. For example, several methods shorten the clearing time by developing different chemical mixtures with high refractive index (RI), such as Sca*l*eS,^[^ 24 ^]^ SeeDB (see deep brain),^[^ 25, 26 ^]^ *Clear^T^*,^[^ 27 ^]^ clearing‐enhanced 3D microscopy,^[^ 28 ^]^ and FOCM.^[^ 29 ^]^ They performed well on embryos, neonatal mouse brains, or thin brain slices, while showed slightly insufficient transparency on whole adult brains.^[^ 30, 31, 32 ^]^ Clear lipid‐exchanged acrylamide‐hybridized rigid imaging/immunostaining/in situ hybridization‐compatible tissue‐hydrogel (CLARITY) and its variants utilize ionic detergents and hydrogel embedding to clear the large tissues and achieve high tissue transparency,^[^ 31, 32, 33, 34, 35, 36 ^]^ but required highly‐specialized devices or cumbersome steps. Clear unobstructed brain imaging cocktails (CUBIC)‐series methods and passive CLARITY technique (PACT) can render the intact adult mouse brains highly transparent by simple incubation with high concentration detergents,^[^ 37, 38, 39, 40, 41 ^]^ while it still required about 1–2 weeks or more for clearing.

Moreover, due to the usage of organic solvents or high concentration detergents, most clearing methods with high clearing capability are incompatible with lipophilic dyes, such as DiI, which has been widely used to trace neuronal structures and vasculatures.^[^ 27, 42, 43, 44, 45 ^]^ Above all, there still lacks a method that can fulfill the following desirable features, including ultrafast clearing speed, high transparency for whole organs, fine compatibility with lipophilic dyes, and ease of use, simultaneously. Addressing all these issues would facilitate more widespread applications of tissue optical clearing techniques.

In this work, we developed a rapid aqueous clearing method based on m‐xylylenediamine (MXDA), which was firstly introduced into tissue clearing, termed the MXDA‐based Aqueous Clearing System (MACS). MACS achieved high transparency of intact organs and rodent bodies in a fairly short time only by simple incubation, and showed ideal compatibility with multiple probes, especially for lipophilic dyes. MACS is applicable for imaging the neural structures of transgenic whole adult brains and immunostained mouse embryos, as well as the neural projections throughout the whole brain labeled by viruses. MACS also allows imaging of DiI‐labeled vascular structures of various organs, including the intact brain, spinal cord, kidney, spleen, heart, and intestine. Using MACS with DiI labeling, we generated the 3D pathology of glomeruli in diabetic kidneys and compared the distribution of glomeruli between normal and diseased kidneys. MACS is expected to be widely used and to promote comprehensive morphological and pathological studies of intact organs.

## 2 Results

### 2.1 Development of MACS for Rapid Clearing of Multiscale Tissues

Urea‐based clearing methods show good clearing performance owing to urea's strong hyperhydration ability, which is derived from two NH_2_ groups.^[^ 12 ^]^ Hyperhydration can dissolve dense fiber proteins and disturb their hydrogen‐bonding networks, leading to reduced light scattering and RI homogenization within tissues. However, urea‐based methods are limited by long incubation time due to the slow hyperhydration speed and low RI of urea solution. We considered to choose new chemicals that could offer highly efficient clearing for various kinds of tissues. MXDA was found to have great potential in tissue clearing. MXDA is a colorless and water miscible liquid with good fluidity and has two NH_2_ groups similar to urea (Figure S1a, Supporting Information), it is expected to have great hyperhydration ability and is easy to penetrate into tissue. Additionally, MXDA has a high RI up to 1.57, which is critical for RI matching in tissue clearing procedure. As excepted, MXDA solution (40 vol%) efficiently rendered 1‐mm‐thick brain sections transparent within 30 min with preservation of signals from enhanced green fluorescent protein (EGFP) (Figure S1b, Supporting Information). Additionally, 30% w/v sorbitol mixed with 40 vol% MXDA provided increased performance in both clearing effect and fluorescence preservation (Figure S1c–h, Supporting Information). We refer to this reagent as MACS‐R1.

However, for large samples such as whole adult mouse brain, individual MACS‐R1 reagents failed to achieve sufficient transparency (Figure S2a top, Supporting Information). We reasoned that when the large samples were only treated with MACS‐R1, the high osmolality might directly make tissue shrink and the clearing reagents would not effectively penetrate into tissue, leading to poor clearing performance. So we introduced MACS‐R0, which has low concentrations of both MXDA and sorbitol (half concentration of MACS‐R1), as the first step of the clearing. It could penetrate quickly into large tissue and lead to high‐performance tissue hyperhydration, making the tissue become swollen and more permeable. We also introduced MACS‐R2 by increasing the sorbitol concentration for higher RI than MACS‐R1. Then, the MACS‐R1 and MACS‐R2 with high RI were used to penetrate throughout the tissue and gradually homogenize the RI within tissue to achieve high transparency. The tissue would gradually return to its original size due to the high osmolality of the two clearing reagents (Figure S2a bottom, Supporting Information).

Hence, taking clearing efficiency and convenience into account, a tri‐step protocol with graded chemical solutions was designed, termed MACS, by sequentially immersing tissue in MACS‐R0 (20 vol% MXDA, 15% w/v sorbitol, RI = 1.40), MACS‐R1 (40 vol% MXDA, 30% w/v sorbitol, RI = 1.48), and finally in MACS‐R2 (40 vol% MXDA, 50% w/v sorbitol, RI = 1.51) for RI matching. This tri‐step protocol is enough for providing impressive clearing performance.

**Figure 1:**
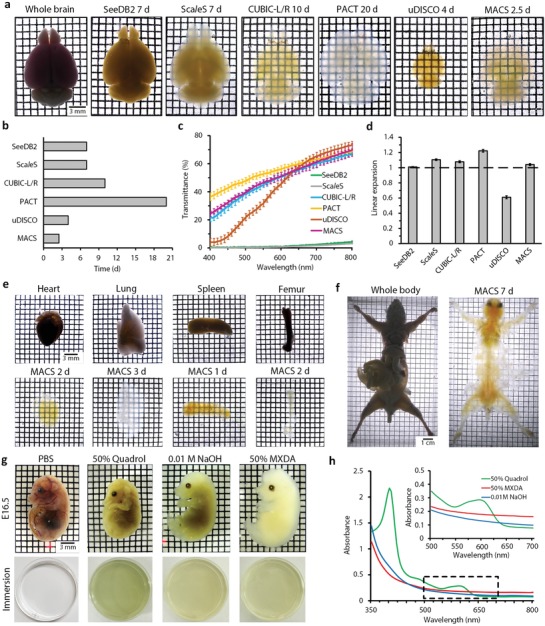
MACS enables rapid clearing for multiscale tissues with decolorization. a) Bright field images of whole adult mouse brains cleared by different clearing protocols. b) Comparison of clearing time needed for each method. c) Transmittance curves of cleared whole brains treated by different methods. Light transmittance was measured from 400 to 800 nm (*n* = 3). d) Quantification of linear expansion of whole brains after clearing by each method (*n* = 3). e) Clearing performance of both hard and soft organs cleared by MACS. f) Clearing performance of MACS for P60 (Postnatal day 60) whole mouse body. g) The decolorization effect of different solutions, including 50 vol% MXDA, 50 vol% Quadrol, and 0.01 m NaOH. The reflection images of fixed embryos and immersions are shown before and after decolorization. Note that each immersion is colorless before decolorization. h) The absorbance curves of each solution were measured after decolorization. Inset: magnification of the boxed region. The values in (c and d) are presented as the mean ± SD.

We established corresponding clearing protocols for different kinds of tissues and organs based on the tri‐step design (Figure S2b–e, Supporting Information). With MACS, we successfully cleared the intact brain with a high level of transparency within only 2.5 days (**Figure** **1**a). Compared with other available clearing methods, MACS renders brain samples highly transparent much faster (Figure 1a–c and Figure S3a,b,d, Supporting Information) and nearly maintains the sample size after transient expansion (Figure 1d and Figure S3c, Supporting Information). The computed tomography (CT) reconstruction images revealed no significant change of brain volume before and after MACS clearing (Figure S4a,b, Supporting Information). The outlines and internal regions of the brain slices overlapped well before and after MACS treatment (Figure S4c–h, Supporting Information). Furthermore, to investigate the influence of MACS on the preservation of fine structures, we imaged a typical pyramidal neuron and single microglia before and after clearing. The results showed that MACS could maintain the cell morphology and fine structures well (Figure S4i,j, Supporting Information).

MACS could also efficiently clarify other mouse tissues, including both soft internal organs and hard bones (Figure 1e), and was also applicable for the adult mouse body (Figure 1f). Additionally, MACS was effective for intact adult rat organs (Figure S5a, Supporting Information). Notably, MACS demonstrated superior ability in clearing mouse embryos and pups (Figure S5b,c Supporting Information).

We also found that MXDA solution could efficiently decolorize heme‐rich tissues, such as embryos (Figure 1g). The absorbance of the decolorizing medium indicated that MXDA decolorized the samples in a manner similar to that of NaOH solution (release Fe) but quite different from that of Quadrol solution (release heme) used in CUBIC (Figure 1h).^[^ 39 ^]^ Additionally, MXDA showed high pH stability over NaOH solution during clearing (Table S1, Supporting Information). The decolorizing capability of MXDA enables MACS to decolorize samples during clarification (Figure S5d, Supporting Information).

### 2.2 MACS Preserves Signals of Multiple Fluorescent Probes, Especially Lipophilic Dyes

**Figure 2:**
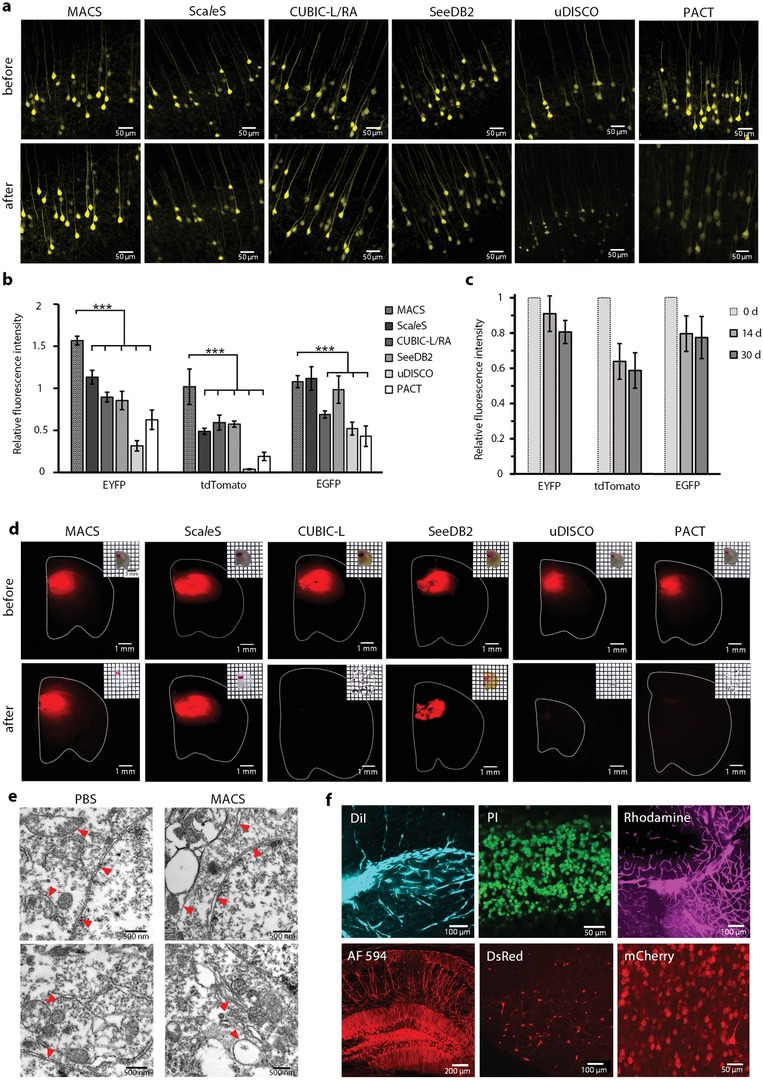
MACS maintains the signals of multiple fluorescent probes. a) Fluorescence images of endogenous EYFP signals (1 mm *Thy1*‐YFP‐H brain slices) before and after MACS clearing compared with other clearing protocols. b) Quantification of fluorescence preservation of EYFP, tdTomato, and EGFP after MACS clearing compared with different methods (*n* = 3). c) Quantitative analysis of long‐term fluorescence preservation of EYFP, tdTomato, and EGFP after MACS clearing (*n* = 3). d) Bright field and fluorescence images of DiI‐labeled brain slices before and after clearing by each method. e) Ultramicroscopic imaging of mouse brain samples restored from MACS or PBS by transmission electron microscopy. Red arrow heads indicate typical membrane structures. f) Fluorescence signals labeled by various chemical fluorescent tracers are finely imaged after MACS clearing, including DiI, PI, tetramethylrhodamine (rhodamine), Alexa Fluor 594 (AF 594)‐conjugated antibody, DsRed, and mCherry. All values are presented as the mean ± SD. Statistical significance in (b) (***, *p* < 0.001) was assessed by one‐way ANOVA followed by the Bonferroni post hoc test.

Furthermore, we investigated the fluorescence preservation of MACS for both endogenous fluorescent proteins and chemical fluorescent tracers. The results demonstrated that MACS could preserve the endogenous enhanced yellow fluorescent protein (EYFP), EGFP, and tdTomato very well after clearing (**Figure** **2**a,b and Figure S6a, Supporting Information). We also imaged MACS‐cleared brain slices during long‐term storage, and the fluorescence intensity remained relatively high after one month (Figure 2c and Figure S6b, Supporting Information).

DiI is a commonly used lipophilic fluorescent dye for neural tracing and vascular labeling. Due to the high concentration of membrane‐removing detergents or organic solvents, CUBIC‐L, PACT, and uDISCO are not compatible with DiI labeling. By contrast, MACS can preserve the fluorescence of DiI fairly well, similar to Sca*l*eS and SeeDB2 (Figure 2d). Indeed, MACS used neither detergents nor organic solvents, thus samples treated by MACS almost maintained membrane integrity, which was crucial for DiI signaling (Figure 2e). The superior compatibility enables visualization of neuronal projections in the DiI‐labeled hippocampus region in mouse brain tissue (Figure 2f). We also tested MACS with other types of chemical tracers, including propidium iodide (PI) for nuclear staining, tetramethylrhodamine‐conjugated dextran for vessel labeling, virus‐delivered proteins (DsRed and mCherry), and fluorophore‐conjugated antibodies in immunostaining (Alexa Fluor 594 (AF 594) and Alexa Fluor 633 (AF 633)) (Figure 2f and Figure S6c,d, Supporting Information). The results showed that MACS maintained the fluorescence signals of all tested tracers well.

Additionally, previous studies demonstrated that CM‐DiI could be used as an alternative in CLARITY‐based methods. CM‐DiI is an aldehyde‐fixably modified version of DiI which adheres not only to the cellular membranes but also protein structures after fixation, such that it would remain in the tissue after lipid extraction.^[^ 46 ^]^ However, we found that the signals from CM‐DiI were only partially remained after treatment by CUBIC‐L, PACT, and uDISCO, the signal loss was obvious as previously reported.^[^ 47 ^]^ Due to the good membrane integrity after MACS treatment, both the DiI and CM‐DiI signals were well maintained without any obvious loss (Figure S6e, Supporting Information).

### 2.3 MACS Achieves 3D Reconstruction of Neural Structures in Intact Tissues

**Figure 3:**
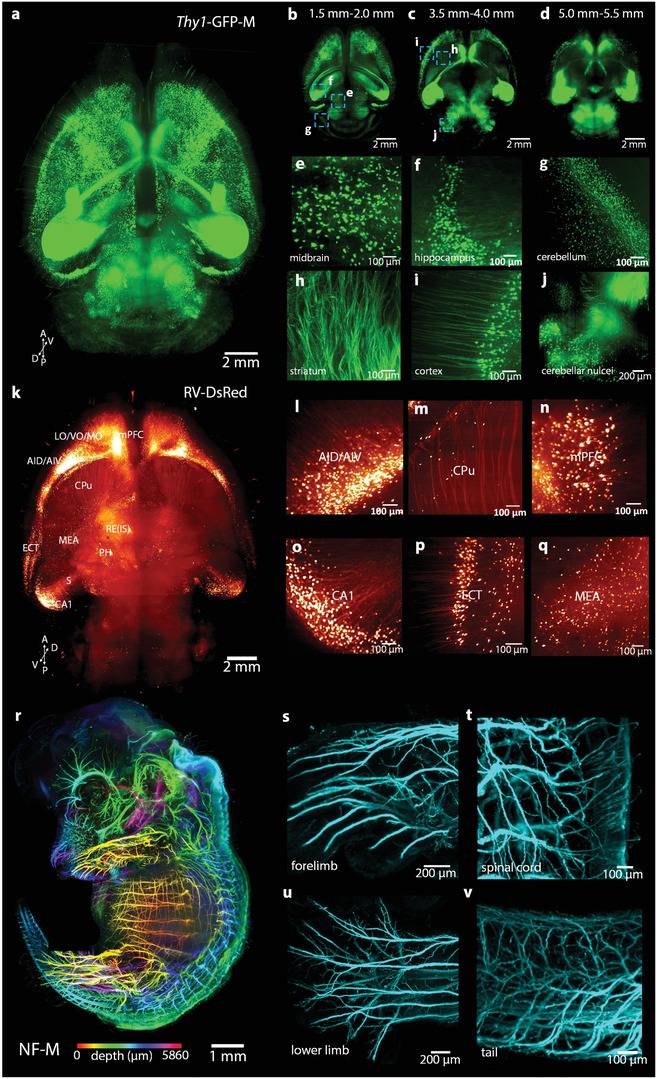
MACS is applicable for 3D imaging and reconstruction of neural structures in intact tissues. a) 3D reconstruction of LSFM images of whole brain (*Thy1*‐GFP‐M) cleared by MACS. Maximum projection of acquired images between b) 1.5–2.0 mm, c) 3.5–4.0 mm, and d) 5.0–5.5 mm. The high‐magnification images of cleared brain reveal fine structures at different positions of the brain, including the e) midbrain, f) hippocampus, g) cerebellum, h) striatum, i) cortex, and j) cerebellar nuclei. k) 3D reconstruction of RV‐labeled afferent projections to nucleus reuniens (RE) throughout the whole brain. Several regions of specific projections to RE, including the l) dorsal/ventral agranular insular cortex (AID/AIV), m) caudate putamen (CPu), n) medial prefrontal cortex (mPFC), o) ventral CA1 of the hippocampus region, p) ectorhinal cortex (ECT), and q) medial amygdaloid nucleus (MEA). r) 3D reconstruction of whole embryo (E14.5) labeled for neurofilament (NF‐M). The images along the z stack are colored by spectrum. Details of the nerve innervation in the s) forelimb, t) spinal cord, u) lower limb, and v) tail. Thin nerve fibers are finely labeled and detected.

Using the rapid MACS clearing protocol, we cleared transgenic whole mouse brains and performed fluorescence imaging by light sheet fluorescence microscopy (LSFM). We obtained the neurons in *Thy1*‐GFP‐M mouse brain at different depths and performed 3D reconstruction (**Figure** **3**a–d and Movie S1, Supporting Information). The fine neural structures were well observed in different brain regions, including the midbrain, hippocampus, cerebellum, striatum, cortex, and cerebellar nuclei (Figure 3e–j). We also cleared and imaged the *Sst*‐IRES‐Cre::Ai14 transgenic mouse brain, and MACS allowed fine imaging of tdTomato fluorescence labeled neurons throughout the whole brain at single‐cell resolution (Figure S7a–f, Supporting Information).

Virus labeling is widely used to reveal neural circuits across the whole brain.^[^ 48, 49 ^]^ Here, we applied MACS protocol to visualize neuronal projections labeled by different types of virus, including retrograde rabies virus (RV) and anterograde adeno‐associated virus (AAV). We imaged the input and output of the nucleus reuniens (RE), which reportedly receives afferent projections across the brain and is an important temporal constraint in hippocampus‐RE‐mPFC circuits.^[^ 50, 51 ^]^ We injected RV‐DsRed and AAV‐mCherry into the RE region for retrograde and anterograde tracing of the projections, respectively (Figure S7g, Supporting Information). The 3D rendering of acquired images showed a diverse and widely distributed set of afferents to RE (Figure 3k), which heavily projected from the dorsal/ventral agranular insular cortex (AID/AIV), medial prefrontal cortex (mPFC), ventral CA1 of the hippocampus, ectorhinal cortex (ECT), medial amygdaloid nucleus (MEA) (Figure 3l–q), etc. Moreover, the 3D rendering showed a relatively limited output from RE, which was mainly directed to the hippocampal formation (e.g., CA1), ventral subiculum (S) and mPFC (Figure S7h–k, Supporting Information), as previously reported.^[^ 52 ^]^

In addition to transgenic labeling and virus labeling, immunostaining is a powerful method to label tissues. Due to the hyperhydration of MXDA used in MACS, we explored whether MXDA pretreatment would enhance the permeability of antibodies in immunostaining. The results revealed that MXDA pretreated samples could achieve deeper staining than those without MXDA pretreatment (Figure S8a–c, Supporting Information). After MXDA pretreatment, whole embryos were stained with anti‐neurofilament antibody and followed by MACS clearing and imaging with LSFM (Figure S8d, Supporting Information). We obtained the 3D nerve distributions of embryos at different ages (Figure 3r and Figure S8e,j, Movie S2, Supporting Information). The fine neural branches in the limbs, spinal cord, tail, and whisker pad could be clearly visualized (Figure 3s–v and Figure S8f–i, Supporting Information).

### 2.4 MACS Enables 3D Mapping of the DiI‐Labeled Vascular System

High‐resolution reconstruction of the vasculatures of various organs facilitates studies of many vascular‐associated diseases.^[^ 53, 54 ^]^ A previous study provided an excellent labeling protocol for vasculatures by perfusion of DiI solution,^[^ 45 ^]^ which has been demonstrated experimentally to be more effective on specific mouse organs (e.g., mouse spleen and kidney) than some common‐used labeling methods (Figure S9, Supporting Information). However, few clearing methods could be applied to this labeling because of incompatibility. Here, due to the superior compatibility with DiI and high clearing capability, we applied MACS to acquire the vasculatures of DiI‐labeled organs, including the whole mouse brain, spinal cord, and other internal organs.

**Figure 4:**
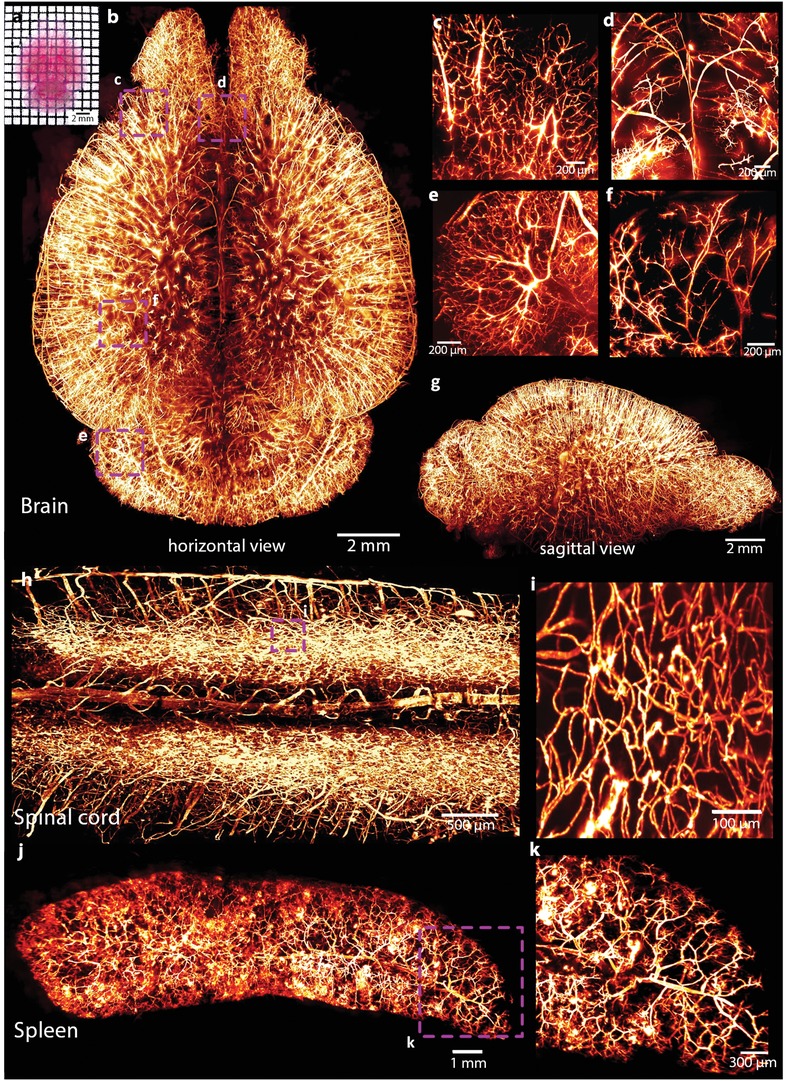
MACS enables 3D visualization of the vascular networks of DiI‐labeled mouse organs. a) Direct view of DiI‐labeled vasculatures in the cleared brain. b) 3D rendering of the vascular network throughout an entire adult brain imaged by LSFM. Detailed vasculature in the c) cortex, d) middle of the brain, e) cerebellum, and f) hippocampus. g) Sagittal view of the reconstructed brain shown in (a). h) 3D rendering of vasculatures in the spinal cord. i) Magnification of boxed region in (h), the surrounded capillaries are finely labeled and clearly visualized. j) Reconstructed vascular network of adult mouse spleen. k) Magnification of boxed region in (j), the small branches are well detected.

After MACS clearing, the DiI‐labeled vasculature of the whole brain could be observed directly (**Figure** **4**a). Combined with LSFM imaging, we visualized the brain vasculature in 3D (Figure 4b and Movie S3, Supporting Information). The detailed vascular structures in the cortex, middle of the brain, cerebellum, and hippocampus could be clearly identified (Figure 4c–f). The sagittal view showed the vascular distribution along the *z*‐axis (Figure 4g). The spinal cord was also finely imaged with both the central blood vessels and the surrounding capillaries distinguished (Figure 4h,i and Movie S4, Supporting Information).

We also acquired 3D vasculatures of other internal organs labeled with DiI, including the spleen, kidney, intestine, and heart. The vascular network throughout the entire spleen was constructed with fine vessels clearly visualized (Figure 4j,k and Movie S5, Supporting Information), and individual glomeruli and attached capillaries throughout the entire kidney could be easily identified and used for quantification (Figure S10a–e, Supporting Information). The imaging results of the intestine showed a rather detailed network of blood vessels in the small intestinal wall and internal intestinal villi (Figure S10f–i, Supporting Information). The blood vessels in an intact heart could also be imaged and reconstructed (Figure S10j, Supporting Information).

### 2.5 MACS Facilitates 3D Pathology of Diabetic Kidneys with DiI Labeling

**Figure 5:**
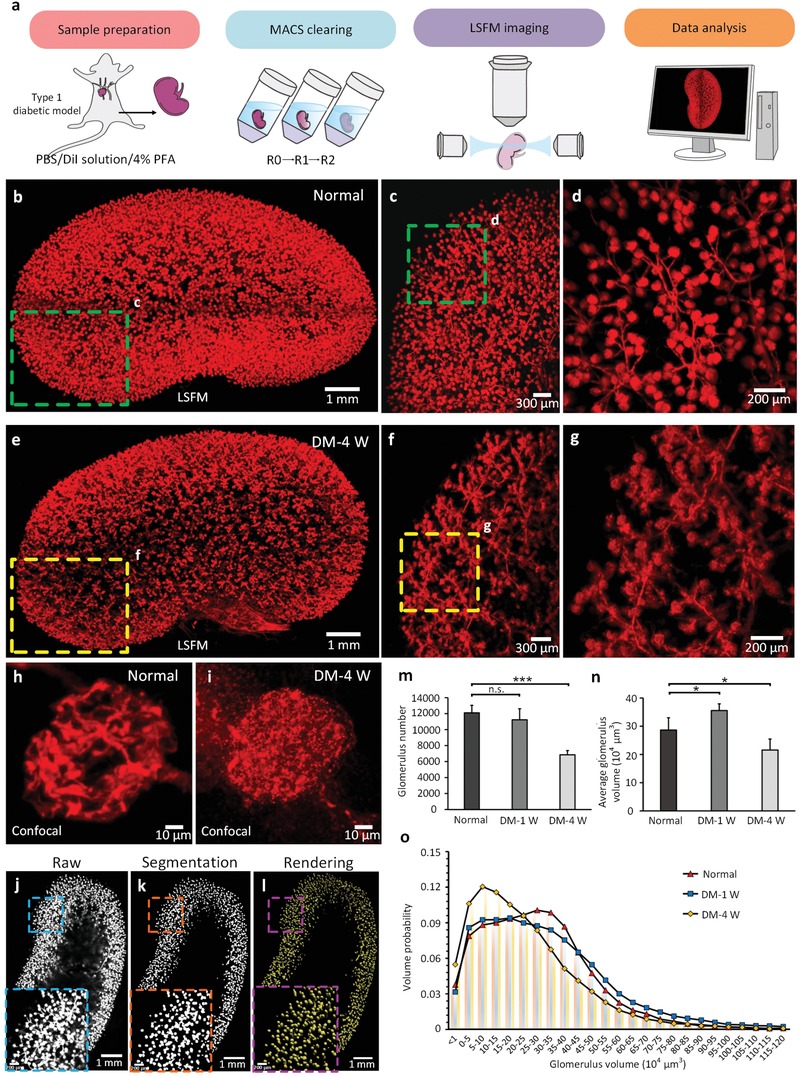
MACS is applicable to the 3D pathology of glomeruli in diabetic kidneys. a) The experimental work flow for labeling, clearing, imaging and quantitative analysis of mouse kidneys. b) 3D reconstruction of glomerular tufts and vessels in a normal kidney by LSFM imaging. c) Magnification of boxed region in (b). d) Magnification of boxed region in (c). e) 3D reconstruction of the distribution of glomerular tufts in a 4 W type 1 diabetic kidney by LSFM imaging. f) Magnification of boxed region in (e). g) Magnification of boxed region in (f). Confocal images of individual glomerular tufts reveal fine capillary structure in h) healthy and i) 4 W diabetic mice. j–l) The pipeline for image processing and counting of glomeruli. Quantitative analysis of glomeruli from normal and diabetic kidneys, including the m) total number of glomeruli, n) glomerular volumes, and o) the probability distribution of tuft volumes. The values in (m and n) are the mean ± SD.; statistical significance in (m and n) (*, *p* < 0.05; ***, *p* < 0.001; n.s., not significant) was assessed by one‐way ANOVA followed by the Bonferroni post hoc test (*n* = 6).

Diabetic nephropathy (DN) is one of the most serious chronic capillary complications of diabetes.^[^ 55 ^]^ Globally, DN mainly causes chronic kidney disease and end‐stage renal disease.^[^ 56 ^]^ The glomerulus is a highly specialized capillary tuft at the proximal part of the nephron, and the main pathology of the vasculature of diabetic kidneys is characterized by a glomerular lesion induced by high blood glucose.^[^ 57 ^]^ Here, we used DiI perfusion to label vascular structures in mouse kidneys, followed by MACS clearing and LSFM imaging, to perform 3D pathology of glomeruli in diabetic kidneys (**Figure** **5**a). The glomerulus trees and branches in healthy kidneys were clearly visible (Figure 5b–d and Movie S6, Supporting Information). We also observed the distribution of glomeruli in kidneys from type 1 diabetic (T1D) mice by LSFM. Kidneys of diabetic models at 4 weeks (DM‐4 W) presented a glomerulus distribution different from that of normal kidneys, and abnormal glomerulus structures were observed in high‐magnification LSFM images (Figure 5e–g and Movie S7, Supporting Information). The confocal images demonstrated the fine structure of the capillary tufts of the glomerulus and showed that the tufts in the 4 weeks diabetic glomerulus were clustered and seriously damaged (Figure 5h,i).

Furthermore, we performed quantification of the glomerulus number and glomerulus volume based on the obtained images. However, it is difficult to extract glomeruli from labeled vascular structures based on the original images (Figure 5j). To address this issue, we first segmented the images to separate the glomeruli from capillaries (Figure 5k) and imported the segmented image stack to Imaris, followed by automatic quantification of the glomeruli across the whole kidney (Figure 5l).

Based on this method, we compared the total number of glomeruli between diabetic and healthy mice. The glomerulus number of healthy kidneys was approximately 12 000 (Figure 5m), consistent with previous studies.^[^ 58, 59, 60 ^]^ The glomerulus number of 1 week diabetic model (DM‐1 W) was not significantly different from that of normal kidneys, while the number of 4 weeks diabetic kidneys was significantly reduced (Figure 5m). For healthy kidneys, the average glomerulus volume was approximately 2.8 × 10^5^ µm^3^, similar to the volume measured by stereology.^[^ 61 ^]^ The average glomerulus volume increased after 1 week of diabetes but decreased after 4 weeks of diabetes (Figure 5n). We also calculated the volume distribution of glomeruli for healthy and diseased kidneys. We observed an increase in the probability of larger glomeruli in 1 week diabetic mice but a decrease in 4 weeks diabetic mice (Figure 5o). This finding revealed that long‐term T1D would lead to both individual tuft defects and glomerular loss. These results reveal that MACS is of great value for 3D visualization and quantitative analysis of kidneys and may facilitate nephrology studies.

## 3 Discussion

For current aqueous‐based clearing methods, the main urgent issue is that methods with good clearing capability usually need rather long incubation time or complex equipment for clearing, and methods with reduced clearing time always show insufficient transparency for large tissues such as whole adult brains. In this article, we solved this problem by presenting MACS, a rapid and effective aqueous‐based optical clearing method scalable for various tissues. MACS can make samples highly transparent in a fairly short time. For instance, MACS requires only 2.5 days to clear a whole adult brain, saving almost 80% of the time needed for CUBIC. MACS also shows ideal compatibility with multiple probes, especially with lipophilic dyes. Combined with LSFM, MACS performed well in 3D reconstruction of neuronal structures in various intact tissues. Due to its superior compatibility with DiI, MACS is applicable to 3D visualization of DiI‐labeled vascular structures of various tissues and allows the 3D pathology of DiI‐labeled glomeruli in normal and diabetic kidneys.

RI matching is one of the main mechanisms of tissue clearing. Generally, to achieve high tissue transparency, some pre‐treatment procedures should be carried out prior to RI matching step. For instance, a thorough dehydration step is needed in solvent‐based clearing methods because many organic solvents used for RI matching are immiscible with water. For aqueous‐based methods, the hyperhydration can reduce light scattering and enhance permeation of RI matching medium by dissolving dense fibers and disturbing their hydrogen‐bonding networks. In this work, MXDA was first introduced to tissue clearing and used as the central reagent in MACS due to its high RI and strong hyperhydration ability. Its superior liquidity and water solubility can also facilitate the penetration of clearing agents. With the advantages above, MXDA can efficiently decrease the RI mismatch in tissue and significantly contribute to the rapid clearing capability of MACS, addressing the problem of long clearing time (weeks to months) of the available urea‐based methods. The addition of sorbitol further enhances transparency and fluorescence preservation. By controlling the concentration of MXDA and sorbitol, the MACS protocol was designed for three steps by passive immersion. Thereinto, MACS‐R0 plays an important role in enhancing the permeability of tissue due to strong hyperhydration, enabling MACS‐R1 and MACS‐R2 quickly penetrate into tissue and gradually homogenize the RI within tissue. This tri‐step protocol offers rather simple, rapid, highly‐efficient clearing for intact organs.

Recently, several groups have introduced novel physical principles and devices to accelerate the CLARITY clearing procedure, such as stochastic electrotransport and ACT‐PRESTO (active clarity technique‐pressure related efficient and stable transfer of macromolecules into organs).^[^ 35, 36 ^]^ The acceleration is very successful that whole brains could be clarified within several days, but requires highly specialized devices. For MACS, a tri‐step incubation with no need of extra equipment makes it rather simple and convenient for researchers to achieve rapid and high‐performance clearing using this method. Additionally, the solvent‐based clearing methods can also achieve high transparency in a relatively short time but often exhibit fluorescence quenching and tissue shrinkage. Recently, the problem of fluorescence quenching has been partially resolved by many groups.^[^ 18, 19, 21 ^]^ However, the tissue shrinkage caused by thorough dehydration is inevitable. MACS could not only nearly maintain the original sample size with fine structures preserved but also achieve high transparency in a fairly short time, it would be beneficial for many related studies.

For some heme‐rich tissues, such as the spleen, kidney, and embryo, residual blood in these tissues will cause severe light absorbance in the visible region (400–600 nm),^[^ 62 ^]^ thus affecting tissue transparency and imaging quality. To overcome this limitation, several effective chemicals showing good decolorizing capability have been used for decolorization in the latest clearing methods.^[^ 19, 22, 39, 40 ^]^ In this study, we found that MXDA also had excellent decolorizing characteristic. The decolorization principle of MXDA is different from that of Quadrol (releasing hemin) used in CUBIC but similar to that of NaOH (releasing Fe).^[^ 39 ^]^ However, during immersion, the pH of NaOH solution showed an obvious decrease, while MXDA showed high pH stability (Table S1, Supporting Information); thus, MXDA solution performs better than NaOH solution. The decolorization effect of MXDA enables MACS to directly extract residual chromophores in tissues, thus leading to efficient clearing without any additional decolorization steps. Furthermore, MACS is expected to be combined with solvent‐based methods, which are often blood‐sensitive, to provide decolorization prior to clearing.

Compatibility with diverse fluorescent labels is also essential for a successful clearing method. MACS shows great compatibility with both endogenous fluorescence proteins and many chemical fluorescent tracers, and is also compatible with virus labeling and immunostaining. We also find that pretreatment with MXDA solution could enhance the permeability of tissues and promote antibody penetration. Moreover, the immunostained samples nearly maintained their original sizes after MACS clearing and could be finely imaged by LSFM (Figure S8d, Supporting Information). This method will provide valuable tool in studies that require whole‐mount immunostaining and imaging for large tissues with minimal size changes.

Lipophilic carbocyanine dyes, such as DiI, have been widely used to label cell membranes and trace neuronal projections in live and fixed tissues.^[^ 27, 42, 43, 44 ^]^ These dyes can also be used to attain fine labeling of the vasculature.^[^ 45 ^]^ However, existing methods with high clearing capability are often incompatible with lipophilic dyes due to the use of high concentrations of detergents or organic solvents. MACS uses neither solvents nor detergents during clearing, so it preserves the membrane architectures that are critical for DiI labeling thus demonstrates ideal compatibility with lipophilic dyes (Figure 2d,e), along with its high clearing performance, MACS enables 3D visualization of DiI‐labeled vascular structures in various intact organs (Figures 4 and 5 and Figure S10, Supporting Information). Notably, the DiI labeling method could offer more detailed vascular information than other commonly used methods in specific organs (Figure S9, Supporting Information). This approach is expected to facilitate the analysis of vasculature networks in specific disease models. Recently, CM‐DiI has been reported to be used as an alternative of DiI in CLARITY method.^[^ 46 ^]^ However, CM‐DiI was experimentally proved to be only partially compatible with methods using high concentration of detergents or organic solvents, such as CUBIC‐L, PACT, and uDISCO. The signal loss was obvious, which was consistent with the result previously reported.^[^ 47 ^]^ For MACS, both the DiI and CM‐DiI signals were well maintained without obvious loss (Figure S6e, Supporting Information). Additionally, the CM‐DiI dye is nearly 100 times more expensive than the common DiI dye (price from Thermo Fisher). It seems not cost‐effective to label the vasculature by perfusing large amount CM‐DiI dyes. So we believe the excellent DiI compatibility along with the high clearing performance is a big advantage for MACS over existing methods.

For aqueous‐based methods, high RI aqueous solutions are often used for final matching with scatters such as lipid or protein. High concentration sugars and polyalcohols are often employed in matching solution. However, most of these solutions have limited RI and high viscosity. Contrast reagents, such as iohexol solution, can achieve high RI, but too costly for common use. In MACS, due to the high RI of MXDA (up to 1.57), the MACS‐R2 has a high RI of 1.51 with low viscosity, which was sufficient for rapid RI matching and LSFM imaging. Additionally, the fluorescence signals are well maintained over time in MACS‐R2 (Figure 2c and Figure S6b, Supporting Information). In fact, the RI of MACS‐R2 could be easily increased by adding extra sorbitol, which may be used for other experimental purpose, such as imaging with oil‐immersion objectives (RI≈1.52). Notably, the price of MACS ingredient is rather cheap for researchers to afford. These findings imply that the MACS‐R2 is hopeful to be used widely as RI matching solution or mounting media in different studies.

As a newly developed clearing protocol, MACS not only maintains the common advantages of aqueous‐based methods, including good fluorescence preservation and fine size maintenance, but also overcomes the limitations of slow clearing speed and insufficient transparency. MACS is also compatible with lipophilic fluorescent dyes. In summary, MACS is a rapid, highly efficient clearing method with robust compatibility. We believe that MACS could provide a valuable alternative for the clearing, labeling, and imaging of large‐volume tissues and facilitate diagnostic studies for pathological diseases. Moreover, MACS has a great potential for 3D pathology of human clinical samples.

## 4 Experimental Section

##### Animals

Animals were housed in a specific‐pathogen‐free animal house under a 12/12 h light/dark cycle with unrestricted access to food and water. Wild‐type (C57BL/6J, 8–12 weeks old), *Thy1*‐GFP‐M (8–10 weeks old), *Thy1*‐YFP‐H (8–10 weeks old), *Sst‐IRES‐Cre*::Ai14 (8–9 weeks old), *Cx3cr1*‐GFP (8–12 weeks old) mice and Sprague‐Dawley rats (8 weeks old) were used in this study. Animals were selected for each experiment based on their genetic background (wild‐type or fluorescence transgenes). All animal experimental protocols were performed under the Experimental Animal Management Ordinance of Hubei Province, P. R. China, and the guidelines from the Huazhong University of Science and Technology. These protocols were approved by the Institutional Animal Ethics Committee of Huazhong University of Science and Technology.

##### Sample Preparation

Adult mice and rats were deeply anaesthetized with a mixture of 2% α‐chloralose and 10% urethane (8 mL kg^−1^) and were transcardially perfused with 0.01 m phosphate buffered saline (PBS, Sigma, P3813) followed by 4% paraformaldehyde (PFA, Sigma‐Aldrich, 158127) in PBS for fixation. The intact brains, bones, and desired organs were excised from the perfused animal bodies. Mouse embryos were collected from anaesthetized pregnant mice. The day on which a plug was found was defined as embryonic day 0.5 (E0.5). All harvested samples were post‐fixed overnight in 4% PFA at 4 °C. Coronal brain sections (1 mm and 2 mm) were sliced using a commercial vibratome (Leica VT 1200 s, Germany).

##### Construction of the Type 1 Diabetic Model

C57BL/6J mice were intraperitoneally administered with 150 mg kg^−1^ alloxan (Sigma, A7413) for 4 days to create a long‐term T1D mouse model. After 4 days, blood sugar levels exceeded 300 mg dl^−1^, which is a commonly used criterion for the diagnosis of diabetes, and consecutive monitoring of blood glucose was performed to ensure the validation of the model. The day when high blood sugar levels were detected in the mice was defined as day 0. The kidneys of diabetic mice were harvested after 1 week and 4 weeks with the vasculature labeled by DiI and were used to investigate the influence of high blood sugar levels on glomeruli.

##### Preparation of MACS Solutions

The MACS protocol contains three solutions. Recipes for each solution were as follows: MACS‐R0 contains 20 vol% MXDA (Tokyo chemical industry, D0127) and 15% (w/v) sorbitol (Sigma, 85529) mixed with distilled water, a solution containing 40 vol% MXDA and 30% (w/v) sorbitol dissolved in 1 × PBS was termed MACS‐R1. MACS‐R2 was prepared as a mixture of 40 vol% MXDA and 50% (w/v) sorbitol in distilled water. Proper heating with a water bath could promote the dissolution of sorbitol. When preparing MACS solutions, latex gloves should be worn to avoid direct contact with chemicals. Though the MXDA is hardly volatile, a ventilated environment is recommended.

##### MACS Clearing Procedure

For passive clearing, fixed samples were serially incubated in 20–30 mL MACS‐R0, MACS‐R1, and MACS‐R2 solutions in 50 mL conical tubes with gentle shaking. The time needed for clearing in each solution depends on the tissue type and thickness (Figure S2b–e, Supporting Information). Hard bones and whole bodies should be incubated first in 0.2 m ethylene diamine tetraacetic acid (EDTA) (Sinopharm Chemical Reagent Co., Ltd, China) for decalcification and then treated with MACS‐R0, MACS‐R1, and MACS‐R2 in succession. The incubation time in final buffer was adjustable by visual inspection for desired transparency. Notably, the temperature during clearing should be kept below 30 °C for better preservation of endogenous fluorescence signals. Heating to 37 °C at the end of each step for approximately 1–3 h is optional for better clearing performance. All other clearing protocols mentioned in this paper, including SeeDB2, Sca*l*eS, CUBIC‐L/R/RA, PACT, and uDISCO, were performed following the original publications.^[^ 18, 24, 26, 40, 41 ^]^

##### Imaging

Fluorescence images of cleared samples (embryos from E12.5 to E16.5, whole adult brains and entire organs) were acquired with a light sheet microscope (Ultramicroscope I, LaVision BioTec, Germany), which was equipped with a sCMOS camera (Andor Neo 5.5, Oxford Instrument, UK) and a macrozoom body (MVX‐ZB10, Olympus, Japan, magnification from 0.63 × to 6.3 ×) with a 2× objective lens (MVPLAPO2X, Olympus, Japan, NA = 0.5, working distance (WD) = 20 mm). Thin light sheets were illuminated from both the right and left sides of the sample, and a merged image was saved. Light‐sheet microscope stacks were acquired using ImSpector (Version 4.0.360, LaVision BioTec, Germany) as 16‐bit grayscale TIFF images for each channel separately.

An inverted laser‐scanning confocal fluorescence microscope (LSM710, Zeiss, Germany) was used to perform fluorescence imaging of brain sections. Samples were placed on a slide with MACS‐R2 and covered with a coverslip to keep tissue submerged in solutions. A 5× objective lens (FLUAR, NA = 0.25, WD = 12.5 mm), 10× objective lens (FLUAR, NA = 0.5, WD = 2 mm), and a 20× objective lens (PLAN‐APOCHROMAT, NA = 0.8, WD = 550 µm) were used for imaging. Zen 2011 SP2 (Version 8.0.0.273, Carl Zeiss GmbH, Germany) software was used to collect data.

Brain slices used for both chemical screening and DiI compatibility investigation were imaged before or after clearing with a fluorescence stereomicroscope (Axio Zoom. V16, Zeiss, Germany) using a 1× long working distance air objective lens (PLAN Z 1×, NA = 0.25, WD = 56 mm). ZEN 2012 (Version 1.1.2.0, Carl Zeiss GmbH, Germany) was used to collect data.

##### Transmission Electron Microscopy

Fixed brain slices were sequentially treated in MACS solutions for 6–8 h each step, or stored in PBS at 4 °C. The treated samples were then restored by washing in 1× PBS at room temperature for 12 h. The samples were post‐fixed with 2.5% glutaraldehyde for several hours, then washed by 0.1 m PBS three times. The samples were then fixed with 1% osmic acid in 0.1 m PBS buffer for 2 h at room temperature, and washed three times in 0.1 m PBS buffer. The fixed samples were then dehydrated with a series of 30%, 50%, 70%, 90%, 100% ethanol. The 1:2 and 1:1 mixtures of epoxy resin and acetone were then sequentially added to infiltrate the blocks for 8–12 h at 37 °C. The blocks were then incubated in pure epoxy resin at 37 °C overnight, and allowed to polymerize at 65 °C for 2 days. Thin sections were obtained using an ultramicrotomy (EM UC7, Leica, Germany). Sections were stained with uranyl acetate and lead acetate, and visualized using a transmission electron microscope (Tecnai G^2^ 20 TWIN, FEI, USA).

##### Measurement of Light Transmittance

The light transmittance of 2 mm thick mouse brain sections were measured with a commercially available spectrophotometer (Lambda 950, PerkinElmer, USA). Cleared samples were placed on two glass slides covered with black tape, and a customized 3 mm × 3 mm slit was opened to obtain the collimated transmitted beam. Transmittance spectra was measured from 400 to 800 nm.

##### Measurement of Tissue Volume

To measure the volume of the whole mouse brain, a customized microcomputed tomography (micro‐CT) was used.^[^ 63 ^]^ The whole mouse brains were imaged by micro‐CT before and after MACS clearing, and reconstructed in 3D. The volume was calculated by Imaris software.

##### Vasculature Labeling

DiI stock solution was prepared by dissolving 30 mg DiI powder (Aladdin, D131225) in 10 mL 100% ethanol and stored in the dark at room temperature. DiI working solution was prepared by adding 200 µL DiI stock solution into 10 mL diluent (0.01 m PBS and 5% (w/v) glucose at a ratio of 1:4). DiI working solution should be freshly made under room lighting. CM‐DiI (Thermo Fisher Scientifc) solution was prepared as 0.01% (w/v). Anaesthetized mice were first perfused with 0.01 m PBS at a rate of 1–2 mL min^−1^ (total 3–4 min) and perfused with 10–15 mL DiI working solution (or CM‐DiI solution) at a rate of 1–2 mL min^−1^ (total 10–15 min). During perfusion with DiI solution, the ears, nose, and palms would turn slightly purple. Finally, the mice were perfused with 4% PFA for fixation. Tissues of interest were harvested and post‐fixed in 4% PFA overnight.

Tetramethylrhodamine dextran was used to label the vasculature. Dextran (70,000 MW, Lysine Fixable, Invitrogen) was diluted in saline at a concentration of 15 mg mL^‐1^ and injected into the tail vein (0.1 mL per animal). After injection, the animals were placed in a warm cage for 15–20 min before standard perfusion steps. Notably, heparin should not be added to PBS used in the prewashing step of the perfusion (use 0.1 m PBS instead), which would result in better labeling of the vasculature.

DyLight 649 conjugated *L. esculentum* (Tomato) lectin (LEL‐Dylight649, DL‐1178, Vector Laboratories) and Alexa Fluor 647 conjugated anti‐mouse CD31 antibody (CD31‐A647, 102416, BioLegend) were also used to label the vasculature. Lectin was diluted in saline to a concentration of 0.5 mg mL^‐1^ and injected into the tail vein (0.1 mL per mouse). The CD31‐A647 antibody (10 to 15 mg) was diluted in saline and injected into the tail vein (total volume of 200 µL). After injection, the animals were placed in a warm cage for 30 min prior to perfusion.

##### Immunostaining

The following primary antibodies were used in this study: anti‐GFP (Abcam, Ab290, dilution 1:500), anti‐neurofilament (NF‐M) (DSHB, 2H3, dilution 1:100), anti‐beta‐tubulin (Servicebio, GB13017‐2, dilution 1:500), and anti‐tyrosine hydroxylase (Servicebio, GB11181, dilution 1:500). Secondary antibodies including Alexa Fluor 594 goat anti‐rabbit IgG (H+L) (1:500 dilution; A‐11037, Life Technologies), Alexa Fluor 633 goat anti‐rabbit IgG (H+L) (1:500 dilution; A‐21070, Life Technologies), and goat anti‐mouse IgG Alexa Fluor 594 (1: 300 dilution; 115‐585‐146, Jackson ImmunoResearch Laboratories, Inc.) were used.

Fixed brain slices were pre‐treated with 50% MXDA solution for 1–2 days and then washed with PBS several times prior to immunostaining. The recovered brain samples and fixed brain slices without pre‐treatment were both blocked in 0.2% PBST (0.2 vol% Triton X‐100 in PBS) containing 6% goat serum at 37 °C for 3–6 h and then subjected to immunostaining with the primary antibodies in 1–2 mL 0.2% PBST containing 3% goat serum albumin and 0.01% (w/v) sodium azide for 2–3 days at room temperature with rotation. The stained samples were then washed with 10 mL 0.2% PBST several times with rotation and immersed in secondary antibodies in 1–2 mL 0.2% PBST containing 3% goat serum albumin and 0.01% (w/v) sodium azide for 2–3 days at room temperature with rotation. The samples were then washed with 10 mL 0.2% PBST several times and stored in PBS at 4 °C prior to clearing.

For samples with large volumes, such as whole embryos, the following iDISCO immunolabeling protocols were used. Fixed samples were treated with 50% MXDA solution for 1–2 days and then washed with PBS several times. The recovered samples were subsequently transferred to 50% methanol in PBS for 1 h, 80% methanol for 1 h, and 100% methanol for 1 h twice. Samples were then bleached with 5% H_2_O_2_ in 20% DMSO/methanol (vol%) at 4 °C overnight. After bleaching, samples were washed with 100% methanol for 1 h twice, 80% and 50% methanol for 1 h, PBS for 1 h twice, and finally 0.2% PBST (0.2 vol% Triton X‐100 in PBS) for 1 h twice. For the immunostaining step, pretreated samples were incubated in 0.2% PBST containing 20% DMSO and 0.3 m glycine at 37 °C overnight, blocked in 0.2% PBST containing 10% DMSO and 6% goat serum at 37 °C for 1 day, washed in PTwH (PBS–0.2% Tween‐20 with 10 mg mL^−1^ heparin) overnight and then incubated with primary antibody dilutions in PTwH containing 5% DMSO, 3% goat serum and 0.01% (w/v) sodium azide at 37 °C with gentle shaking on a shaker for 5–7 days. Samples were then washed for 2 days with PTwH and then incubated with secondary antibodies diluted in PTwH containing 3% goat serum and 0.01% (w/v) sodium azide at 37 °C with gentle shaking for 5–7 days. Sections were finally washed in PTwH for 2 days and stored in PBS at 4 °C prior to clearing.

##### Neuronal Tracing by RV and AAV

In this study, we used RV‐N2C (G)‐ΔG‐DsRed (RV‐DsRed, BrainVTA, R03002), rAAV‐hSyn‐mCherry‐WPRE‐pA (AAV‐mCherry, BrainVTA, PT‐0100) for tracing neuronal projections. For the RV and AAV injection to RE, 8–9 weeks old C57BL/6J mice were used. The injection site of RV‐DsRed (400 nl) and AAV‐mCherry (400 nl) was targeted to the RE in different mice with the following coordinates: bregma, −0.82 mm; lateral, −0.27 mm; and ventral, 1.75 mm.

For injection, a cranial window was created on the skull to expose the brain area targeted for tracing neurons. The virus was injected into the brain using a custom‐established injector fixed with a pulled glass pipette. The animal was placed in a warm cage after injection for waking up and then transferred into a regular animal room. The animals were kept for 7 days after RV and 28 days after AAV injection before perfusion.

##### Image Data Processing

All raw image data were collected in a lossless TIFF format (8‐bit images for confocal microscopy and 16‐bit images for light sheet microscopy). Processing and 3D rendering were executed by a Dell workstation with 8 core Xeon processor, 256 GB RAM, and Nvidia Quadro P2000 graphics card. We used Imaris (Version 7.6, Bitplane AG) and Fiji (Version 1.51n) for 3D and 2D image visualization, respectively. Stitching of tile scans from light sheet microscopy was performed via Matlab (Version 2014a, Mathworks). The 16‐bit images were transformed to 8‐bit images with Fiji to enable fast processing using different softwares.

##### Quantifications: Measurement of Linear Size Changes

For the measurement of sample expansion and shrinkage, 2 mm brain slices and whole brains were used, and bright field images were taken before and after clearing. Based on top view photos, the area of samples was determined using the ‘polygon‐selections' function of Fiji. The linear expansion value was determined by the square root of the area size change.

##### Quantifications: Relative Fluorescence Quantification

For evaluation of relative fluorescence intensity, the cell body of a neuron was encircled by the ‘freehand‐selection' tool in Fiji, and the mean fluorescence intensity and area were measured. The multiplication values of the two parameters were identified as the total fluorescence intensity of the neurons. The total fluorescence intensity was normalized to intensity in PBS (100%) for the same neuron, which was defined as relative fluorescence.

##### Quantifications: Analysis of Glomerular Number and Volume in the Normal and Diabetic Kidneys

First, each z‐layer of raw 16‐bit LSFM data was normalized to 8 bit with a maximum value of each layer equal to 255. Then, the 8‐bit data were processed by morphological opening with a disk of radius:

(1) $$\text{r}\;=\frac{r_{\text{max}}}{s_{\text{xy}}}$$

where *r*_max_ refers to the maximally allowed radius of glomeruli and was set to 90 µm, and *s*_xy_ denotes the voxel size in the *x*‐*y*‐plane. This process resulted in an image where all objects with sizes less than *r*_max_ were removed. Then, the morphologically opened image was subtracted from the initial image to correct the uneven background. In the resulting image, only objects with sizes less than *r*_max_ were retained. Then, Otsu thresholding was performed to create a binary mask for glomerular information. Next, morphological opening was performed again with a disk of radius:

(2) $$\text{r}\;=\frac{r_{\text{min}}}{s_{\text{xy}}}$$

where *r*_min_ corresponds to the minimal allowed radius and was set to 20 µm. In the output image, only objects with a radius greater than 0.5 × *r*_min_ were preserved. Then, the output binary data were multiplied with the initial 8‐bit data, which extracted most of the vessel information. The segmentation procedure was partly referred to in ref. ^[^ 20 ^]^. The processed data were then imported into Imaris for the next step. The glomerular number and volume were calculated via the surface function of Imaris. The size of the automatically selected surface was manually adjusted to the actual glomerular size by changing the threshold (absolute intensity) parameter, and the remaining vessel structures were manually removed.

##### Statistical Analysis

Data are presented as the mean ± SD and were analyzed using SPSS software (Version 22, IBM, USA) with 95% confidence interval. Sample sizes are indicated in the figure legends. For analysis of statistical significance, the normality of the data distribution in each experiment was checked using the Shapiro–Wilk test. The variance homogeneity for each group was evaluated by Levene's test. *p* values were calculated using an independent‐sample *t*‐test (two‐sided) to compare data between two groups in Figure S4b, Supporting Information. *p* values were calculated using one‐way ANOVA followed by the Bonferroni post hoc test to compare data in Figures 2b and 5m,n and Figure S1g, Supporting Information. In this study, *p* < 0.05 was considered significant (*, *p* < 0.05; **, *p* < 0.01; ***, *p* < 0.001).

## Conflict of Interest

The authors declare no conflict of interest.

## Supporting information

Supporting Information

Supplemental Movie 1

Supplemental Movie 2

Supplemental Movie 3

Supplemental Movie 4

Supplemental Movie 5

Supplemental Movie 6

Supplemental Movie 7
