# Supplementary material for: MACS: Rapid Aqueous Clearing System for 3D Mapping of Intact Organs
Source: Adv Sci (Weinh). 2020 Feb 25;7(8):1903185. doi: 10.1002/advs.201903185 (PMC7175264; doi:10.1002/advs.201903185)
Supplement: Supplementary file 1 — Supporting Information [file ADVS-7-1903185-s001.pdf]

Copyright WILEY-VCH Verlag GmbH & Co. KGaA, 69469 Weinheim, Germany, 2020.

ADVANCED SCIENCE
Open Access

Supporting Information

for *Adv. Sci.,* DOI: 10.1002/advs.201903185

MACS: Rapid Aqueous Clearing System for 3D Mapping of Intact Organs

*Jingtan Zhu, Tingting Yu, Yusha Li, Jianyi Xu, Yisong Qi, Yingtao Yao, Yilin Ma, Peng Wan, Zhilong Chen, Xiangning Li, Hui Gong, Qingming Luo, and Dan Zhu**

Supporting Information

MACS: Rapid aqueous clearing system for 3D mapping of intact organs

Jingtan Zhu, Tingting Yu, Yusha Li, Jianyi Xu, Yisong Qi, Yingtao Yao, Yilin Ma, Peng Wan, Zhilong Chen, Xiangning Li, Hui Gong, Qingming Luo, Dan Zhu*

J. Zhu, Dr. T. Yu, Y. Li, J. Xu, Dr. Y. Qi, Dr. Y. Yao, Y. Ma, P. Wan, Z. Chen, Prof. X. Li, Prof. H. Gong, Prof. Q. Luo, Prof. D. Zhu

Britton Chance Center for Biomedical Photonics, Wuhan National Laboratory for Optoelectronics, Huazhong University of Science and Technology, Wuhan 430074, China

J. Zhu, Dr. T. Yu, Y. Li, J. Xu, Dr. Y. Qi, Dr. Y. Yao, Y. Ma, P. Wan, Z. Chen, Prof. X. Li, Prof. H. Gong, Prof. Q. Luo, Prof. D. Zhu

MoE Key Laboratory for Biomedical Photonics, Huazhong University of Science and Technology, Wuhan 430074, China

*Author for correspondence

Email: dawnzh@mail.hust.edu.cn

This PDF file includes:

Figures S1 to S10
Table S1
Legends for Movies S1 to S7

Other supporting information for this manuscript include the following:

Movies S1 to S7

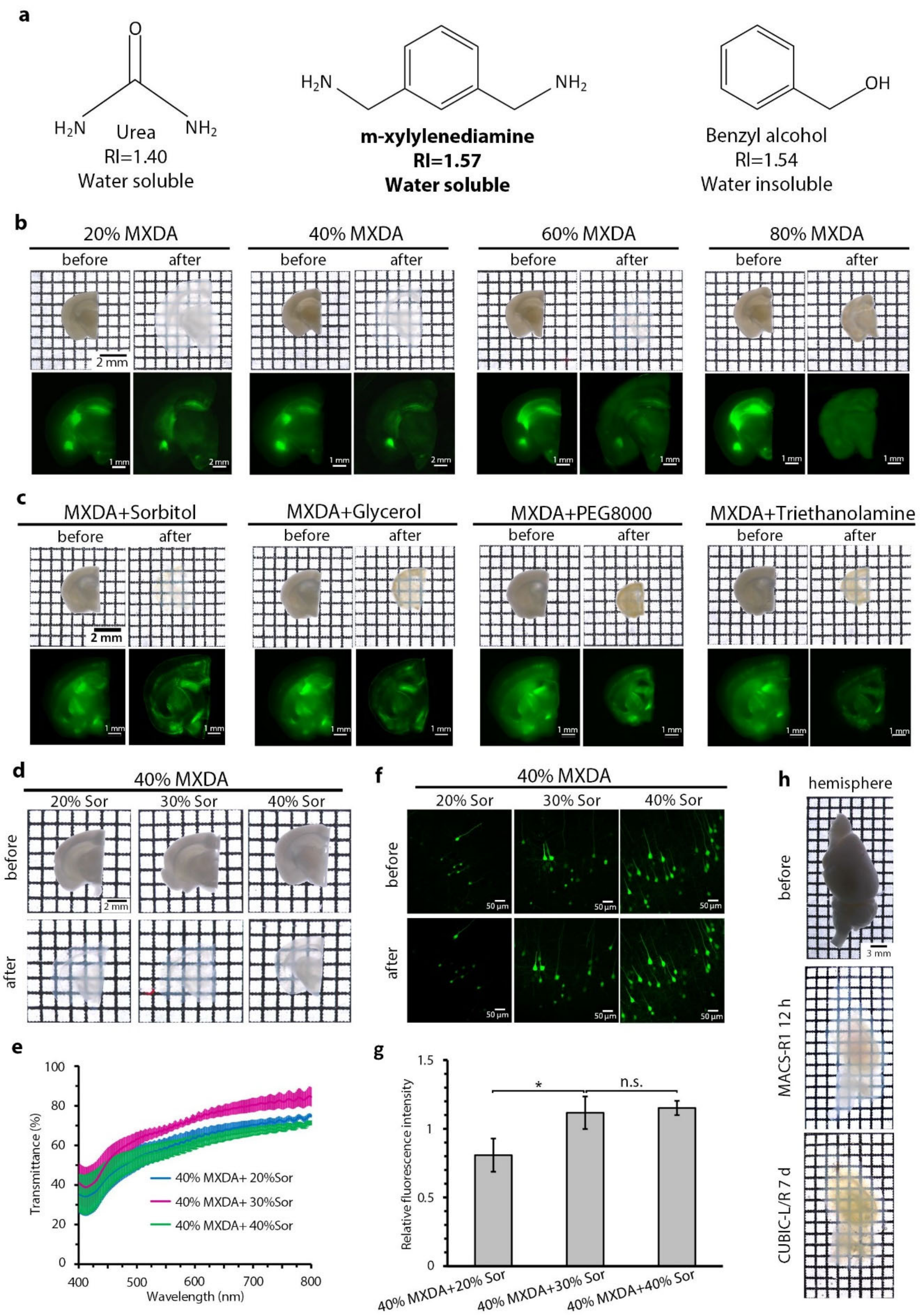
a
Urea
RI=1.40
Water soluble
m-xylylenediamine
RI=1.57
Water soluble
Benzyl alcohol
RI=1.54
Water insoluble
b
20% MXDA
40% MXDA
60% MXDA
80% MXDA
before
after
c
MXDA+Sorbitol
MXDA+Glycerol
MXDA+PEG8000
MXDA+Triethanolamine
d
40% MXDA
20% Sor
30% Sor
40% Sor
e
Transmittance (%)
Wavelength (nm)
40% MXDA+ 20%Sor
40% MXDA+ 30%Sor
40% MXDA+ 40%Sor
f
40% MXDA
g
Relative fluorescence intensity
n.s.
40% MXDA+20% Sor
40% MXDA+30% Sor
40% MXDA+40% Sor
h
hemisphere
MACS-R1 12 h
CUBIC-L/R 7 d

30
31

Figure S1. Determination of reagents for efficient clearing and fluorescence preservation. (**a**) Chemical structures and properties of MXDA compared to those of urea and typical organic solvent (Benzyl alcohol). (**b**) *Thy1*-GFP-M half brain slices (1 mm) were incubated with 20 vol%, 40 vol%, 60 vol% or 80 vol% MXDA solution for 30 min. The bright field and fluorescence images were taken before and after clearing in each solution. (**c**) Sorbitol, glycerol, PEG8000 and triethanolamine were mixed with MXDA. The concentration of MXDA was 40 vol% according to **b**, and the concentration of sorbitol, glycerol, PEG8000 and triethanolamine was 30% (wt/vol). First, 1 mm half brain slices were incubated in each mixture for 30 min. The bright field and fluorescence images were taken before and after immersion in each mixture. (**d**) *Thy1*-GFP-M half brain slices (1 mm) were cleared by 20%, 30% and 40% (wt/vol) sorbitol with 40 vol% MXDA. (**e**) The transmittance curves of samples cleared by each group (n=3). (**f**) Confocal images of EGFP signals of samples cleared by the three solutions. (**g**) Quantification of the relative fluorescence intensity for the EGFP signal after clearing. (**h**) Hemispheres were cleared by MACS-R1 and original CUBIC protocols, respectively. All values are presented as the mean ± s.d.. Statistical significance in **g** (*, $P < 0.05$) was assessed by one-way ANOVA followed by the Bonferroni post hoc test.

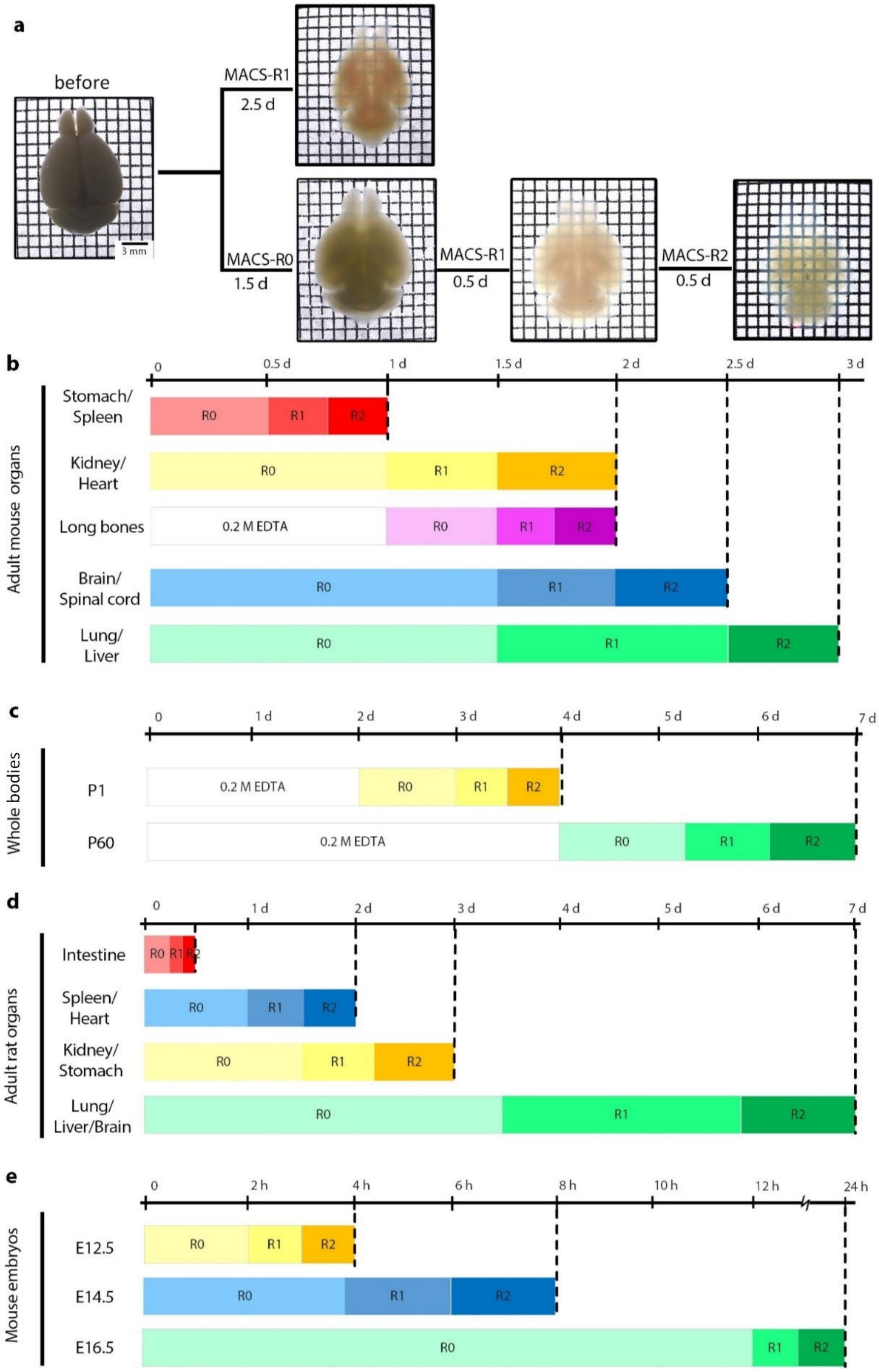
a
before
3 mm
MACS-R1
2.5 d
MACS-R0
1.5 d
MACS-R1
0.5 d
MACS-R2
0.5 d
b
0
0.5 d
1 d
1.5 d
2 d
2.5 d
3 d
Adult mouse organs
Stomach/ Spleen
Kidney/ Heart
Long bones
Brain/ Spinal cord
Lung/ Liver
R0
R1
R2
0.2 M EDTA
c
0
1 d
2 d
3 d
4 d
5 d
6 d
7 d
Whole bodies
P1
P60
d
Adult rat organs
Intestine
Spleen/ Heart
Kidney/ Stomach
Lung/ Liver/Brain
e
0
2 h
4 h
6 h
8 h
10 h
12 h
24 h
Mouse embryos
E12.5
E14.5
E16.5

Figure S2. Tri-step MACS protocol for high-performance clearing. (**a**) Introducing MACS-R0 and MACS-R2 for high performance clearing. Whole adult brains were cleared by individual MACS-R1 and the entire MACS procedure, MACS-R1 alone failed to make whole mouse brain transparent, while the brain was rendered highly transparent using the tri-step protocol. (**b** to **e**) MACS clearing protocol for different tissues, including adult mouse organs, whole mouse bodies, adult rat organs and embryos. For hard tissues or whole bodies, the decalcification procedures with EDTA were introduced and performed at 37°C with gently shaking. The time of each step could be adjustable based on the real-time clearing performance.

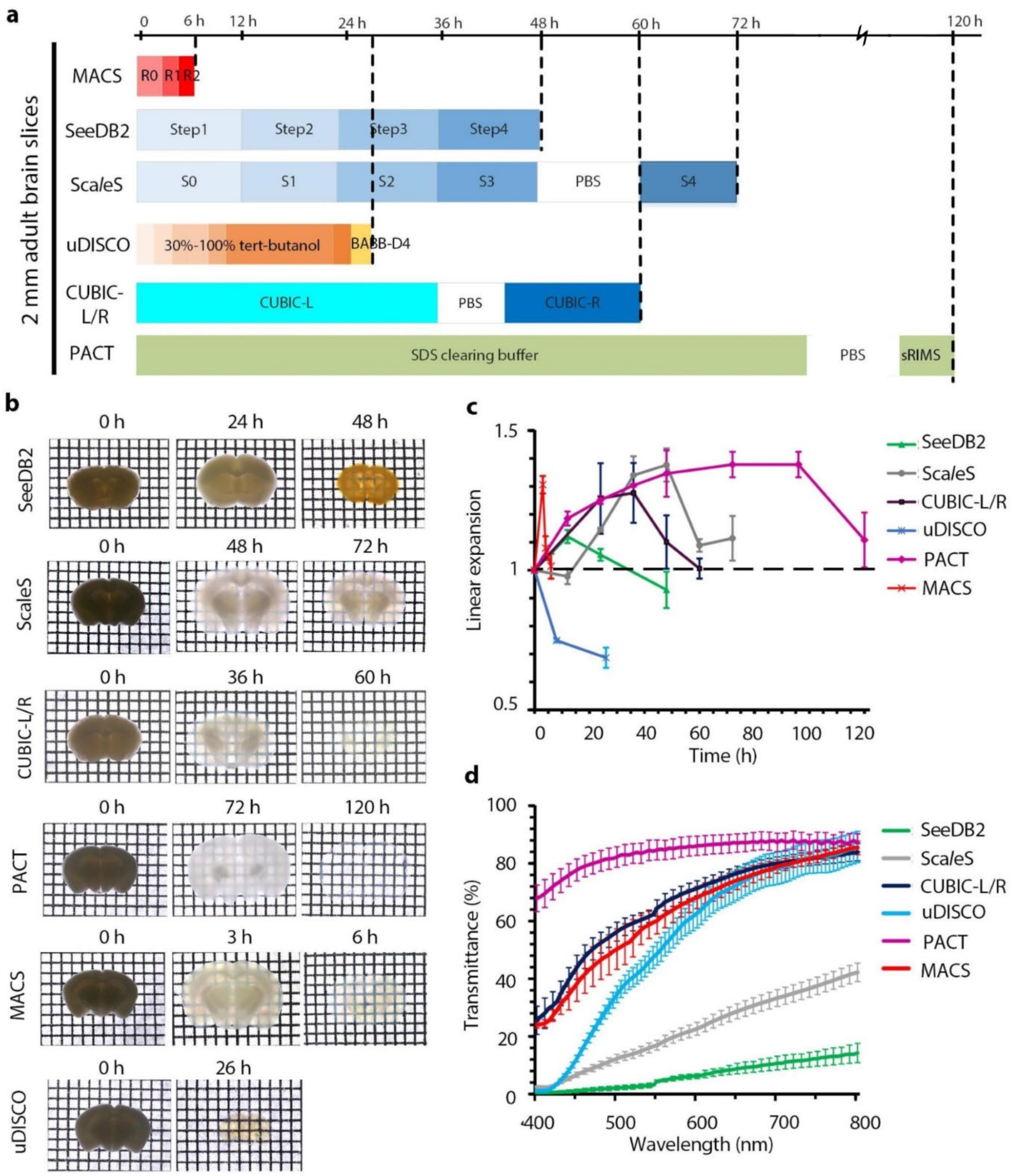

Figure S3. Clearing performance on 2-mm-thick brain slices for different methods. (**a**) Schedules of clearing by MACS, SeeDB2, Sca*l*eS, CUBIC-L/R, PACT and uDISCO on 2 mm adult brain slices. (**b**) Bright field images of samples before, during and after clearing for each protocol. (**c**) Quantification of sample size changes in 2 mm adult slices (n=3) during different optical clearing procedures. (**d**) Transmission curves of 2 mm adult slices (n=3) cleared with various clearing protocols. All values are presented as the mean ± s.d.

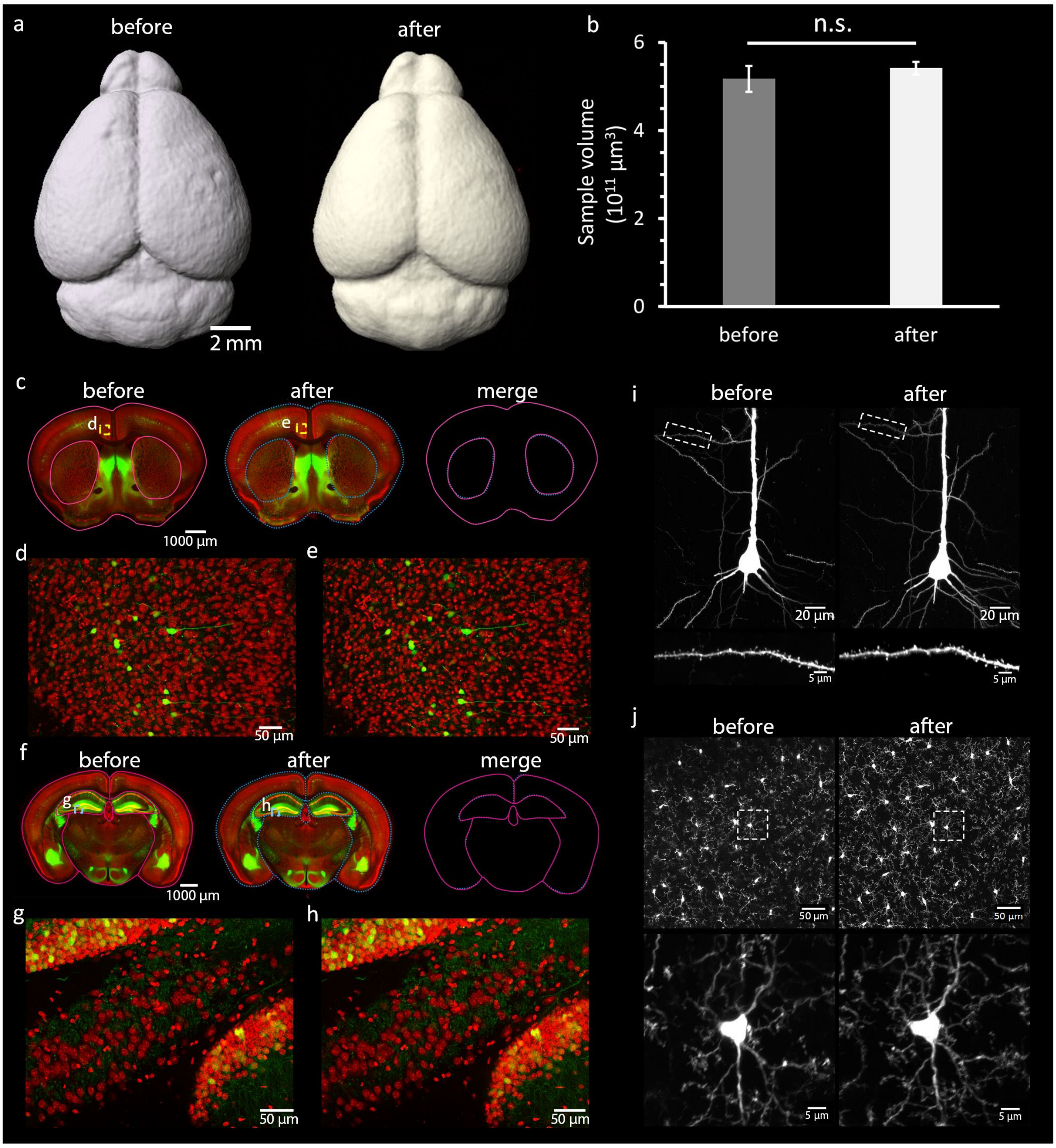
a
before
after
2 mm
b
n.s.
Sample volume
(10^{11} μm^3)
0
2
4
6
before
after
c
before
after
merge
d
e
1000 μm
d
e
50 μm
50 μm
f
before
after
merge
g
h
1000 μm
g
h
50 μm
50 μm
i
before
after
20 μm
20 μm
5 μm
5 μm
j
before
after
50 μm
50 μm
5 μm
5 μm

Figure S4. Preservation of tissue sizes and fine cell structures of brain samples after MACS treatment. (**a**) CT reconstruction of intact mouse brain before and after MACS clearing. (**b**) Measurement of volume before and after MACS clearing. (**c**) Fluorescence images of coronal brain slices (1 mm, Bregma ≈ +0.50 mm) from adult *Thy1*-GFP-M mouse before and after MACS clearing. (**d** and **e**) Magnified images of boxed regions in **c**. (**f**) Fluorescence images of coronal brain slices (1 mm, Bregma ≈ -1.70 mm) before and after MACS clearing. (**g** and **h**) Magnified images of boxed regions in **f**. The borders of the main structures were traced and colored magenta and blue, respectively. (**i**) Typical pyramidal neurons were imaged before and after MACS clearing. (**j**) Microglial cells were imaged before and after MACS clearing. Statistical significance in **b** (n.s., $p>0.05$) was assessed by an independent-sample t test (n=3).

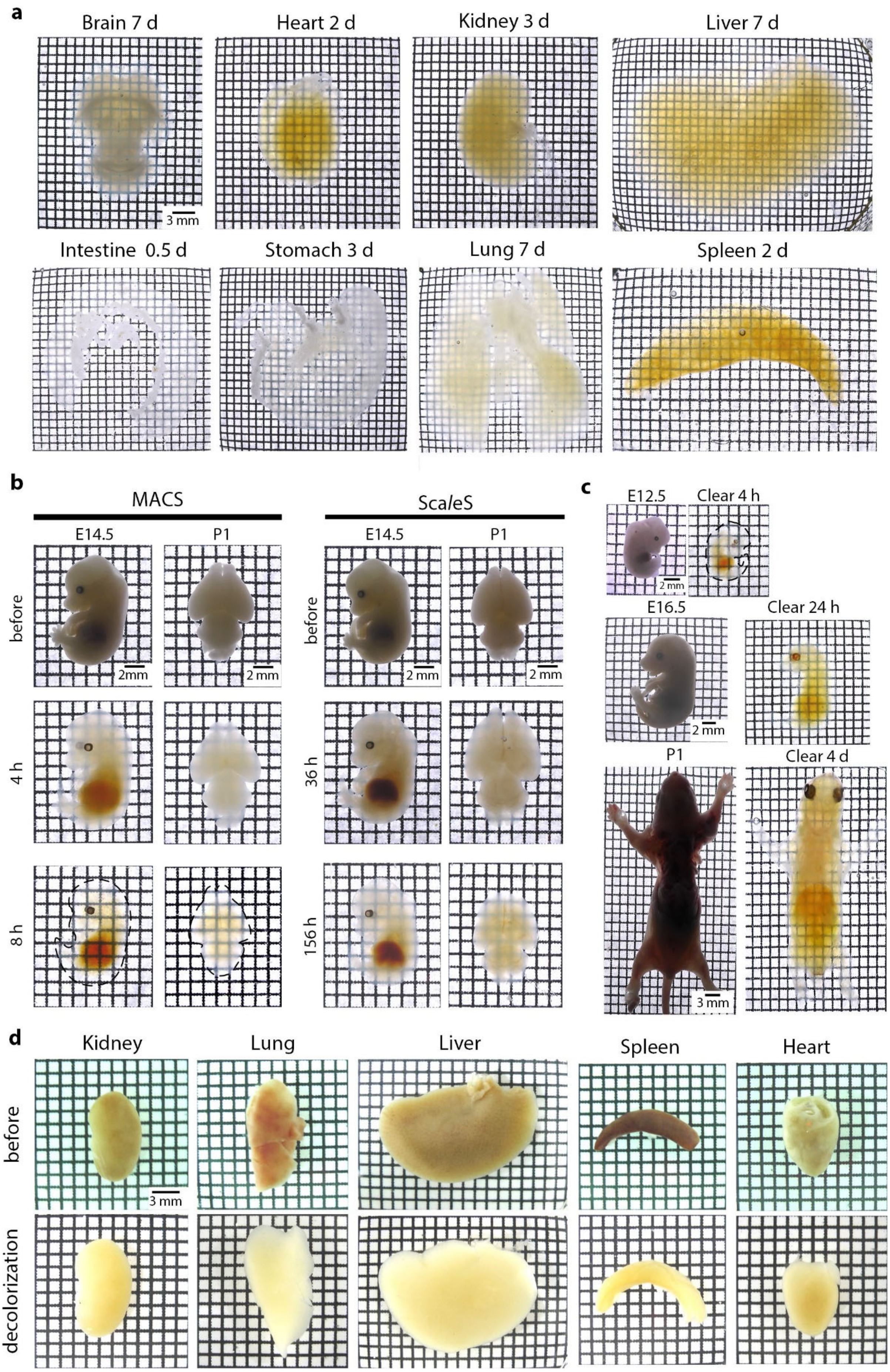
a
Brain 7 d
Heart 2 d
Kidney 3 d
Liver 7 d
3 mm
Intestine 0.5 d
Stomach 3 d
Lung 7 d
Spleen 2 d
b
MACS
ScaleS
E14.5
P1
E14.5
P1
before
2mm
2 mm
2 mm
2 mm
4 h
36 h
8 h
156 h
c
E12.5
Clear 4 h
2 mm
E16.5
Clear 24 h
2 mm
P1
Clear 4 d
3 mm
d
Kidney
Lung
Liver
Spleen
Heart
before
3 mm
decolorization

78

Figure S5. Efficient clearing and decolorization of different tissues by MACS. (**a**) Intact rat organs were cleared with MACS, and bright field images showed that the brain, heart, kidney, liver, intestine, stomach, lung and spleen were rendered optically transparent at the indicated time. (**b**) Comparison of clearing performance for embryos and neonatal mouse brains by Sca*l*eS and MACS. (**c**) Efficient clearing for embryos (E12.5 and E16.5) and P1 pups by MACS. (**d**) Decolorization of different mouse organs by MACS-R0. Heme-rich mouse organs such as kidney, lung, liver, spleen and heart were incubated in MACS-R0 for 24 h and then washed with PBS. Reflection images were taken before and after treatment.

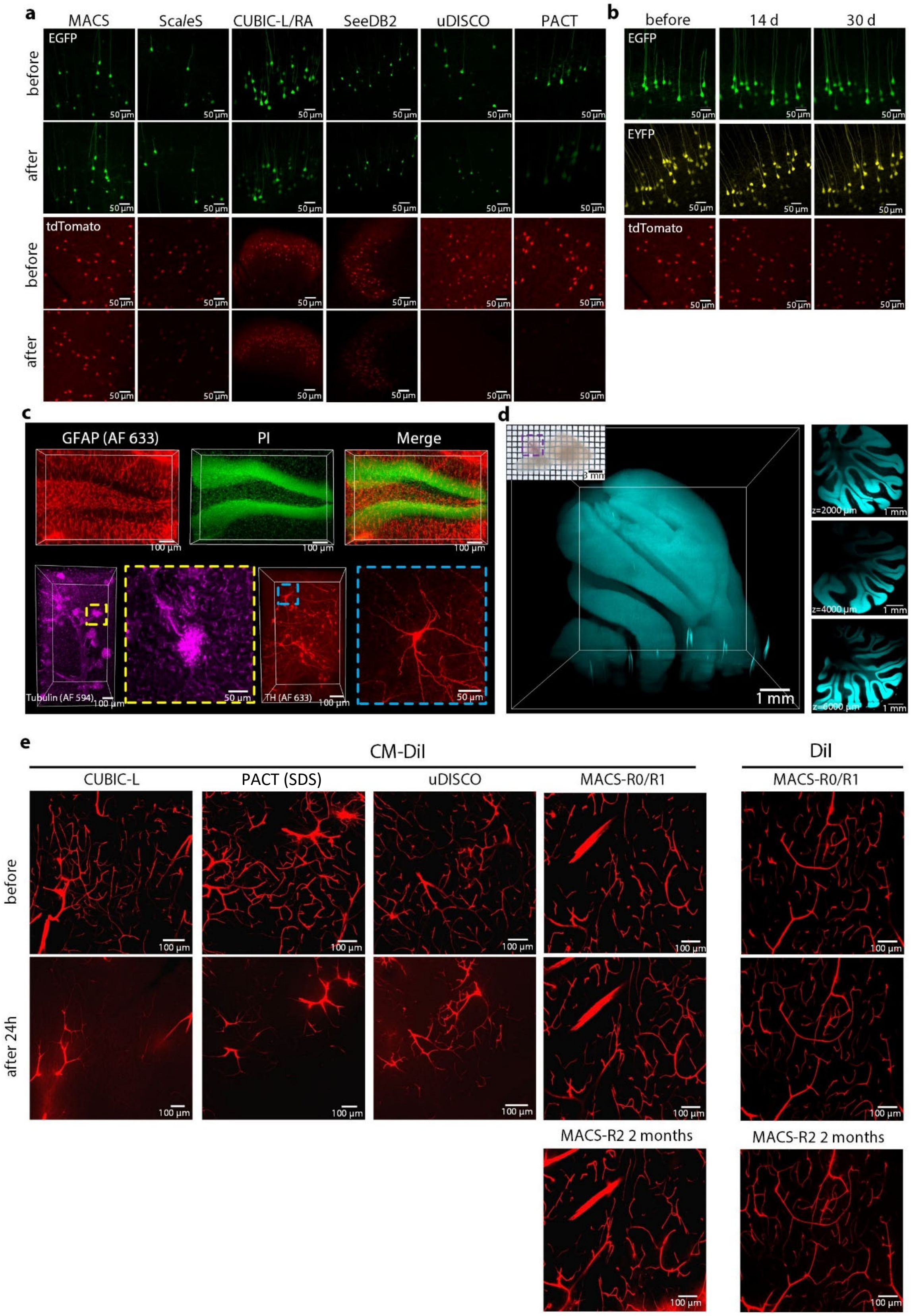
a
MACS
ScaleS
CUBIC-L/RA
SeeDB2
uDISCO
PACT
EGFP
tdTomato
before
after
50 µm
b
before
14 d
30 d
EGFP
EYFP
tdTomato
c
GFAP (AF 633)
PI
Merge
100 µm
Tubulin (AF 594)
TH (AF 633)
50 µm
d
1 mm
z=2000 µm
z=4000 µm
e
CM-DiI
DiI
CUBIC-L
PACT (SDS)
uDISCO
MACS-R0/R1
before
after 24h
MACS-R2 2 months
100 µm

Figure S6. MACS performs good compatibility with transgenic labeling, immunolabeling, nucleus staining and DiI labeling, related to Figure 2. (**a**) Fluorescence images of endogenous EGFP and tdTomato in 1 mm brain slices before and after clarification by each method. (**b**) Fluorescence images of endogenous EGFP, EYFP and tdTomato of MACS-cleared 1 mm brain slices over time. (**c**) (**top**) GFAP immunostaining and PI labeling of 1 mm coronal brain sections. Images show labeling of astrocytes and cell nuclei in hippocampus region. (**lower left**) 3D rendering of 1 mm mouse kidney slice stained by anti-beta tubulin. Magnification of boxed region reveals fine labeling of microtubules. (**lower right**) 3D rendering of 1 mm brain block stained by anti-tyrosine hydrogenase (TH). Magnification of boxed region reveals the structure of an individual TH-positive neuron. (**d**) LSFM imaging and reconstruction of the cerebellum of a PI-stained rat hemisphere cleared by MACS, the imaging depth is over 6800 μm. Optical sections are shown at different depths. The structure of the cerebellum is clearly visible throughout the entire imaging depth. (**e**) Confocal images of CM-DiI labeled brain slices before and after 24 h clearing by each method. MACS could not only preserve the DiI signal, but also preserve the signal from CM-DiI.

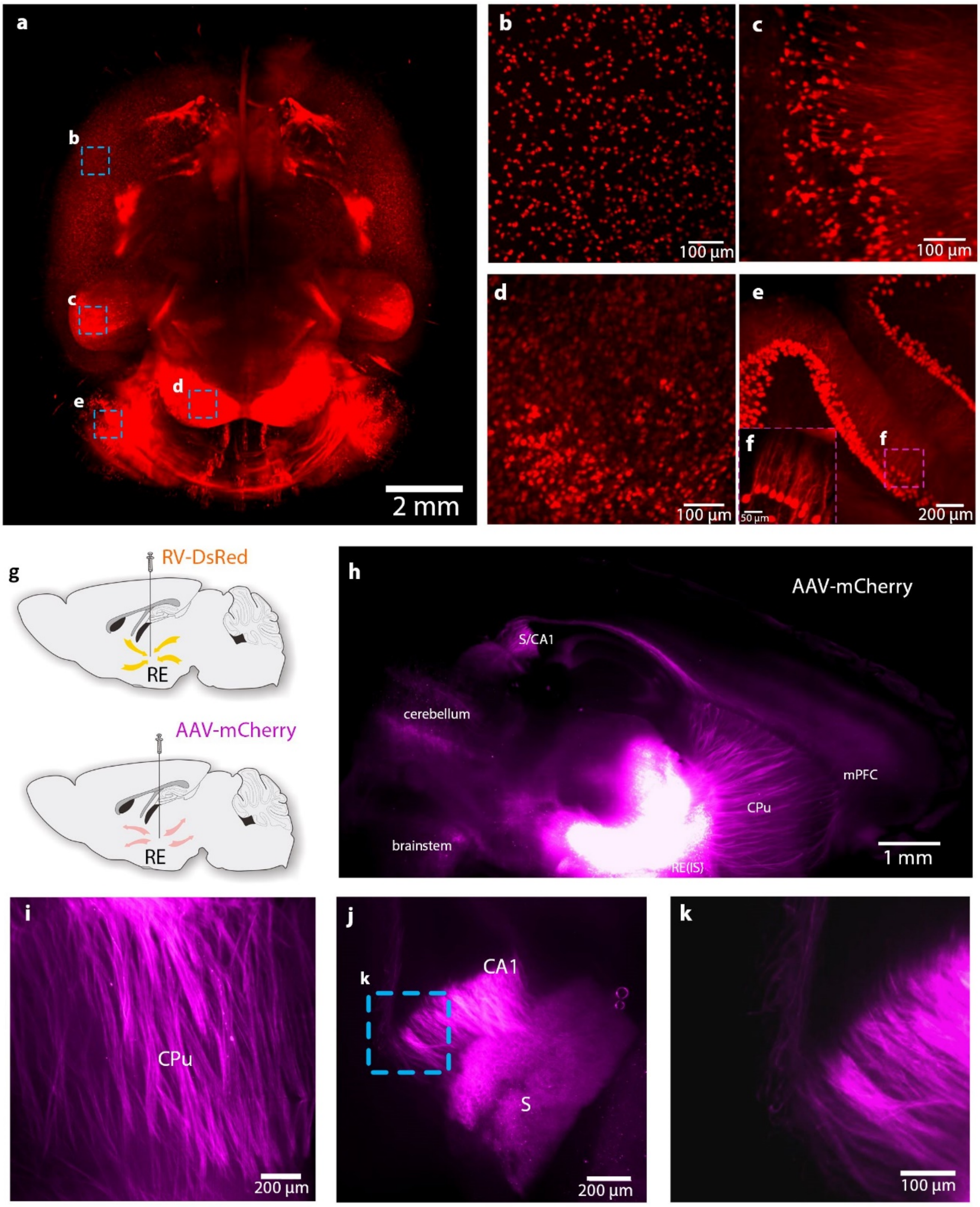
a
b
c
d
e
2 mm
b
100 µm
c
100 µm
d
100 µm
e
f
f
50 µm
200 µm
g
RV-DsRed
RE
AAV-mCherry
RE
h
AAV-mCherry
S/CA1
cerebellum
mPFC
CPu
brainstem
RE(IS)
1 mm
i
CPu
200 µm
j
k
CA1
S
200 µm
k
100 µm

104

105

Figure S7. LSFM imaging of tdTomato-labeled and AAV-injected mouse brains cleared by MACS. (**a**) 3D reconstruction of LSFM images of a tdTomato-labeled (Sst-IRES-Cre::Ai14) whole brain cleared by MACS. (**b** to **e**) High-magnification images of tdTomato-positive neurons in the cortex (**b**), hippocampus (**c**), inferior colliculus (**d**) and cerebellum (**e**). (**f**) Details of the boxed region in **e**, showing the fine neuronal structures at single cell resolution. (**g**) Experimental design for RV and AAV injections. (**h**) 3D reconstruction of AAV-labeled projections from RE in the hemisphere. (**i**) Neural fibers projecting from RE to mPFC via CPu. (**j**) Projections to ventral CA1 of the hippocampus region and ventral subiculum (S). (**k**) The tiny fibers in **j** could be imaged. IS: injection site.

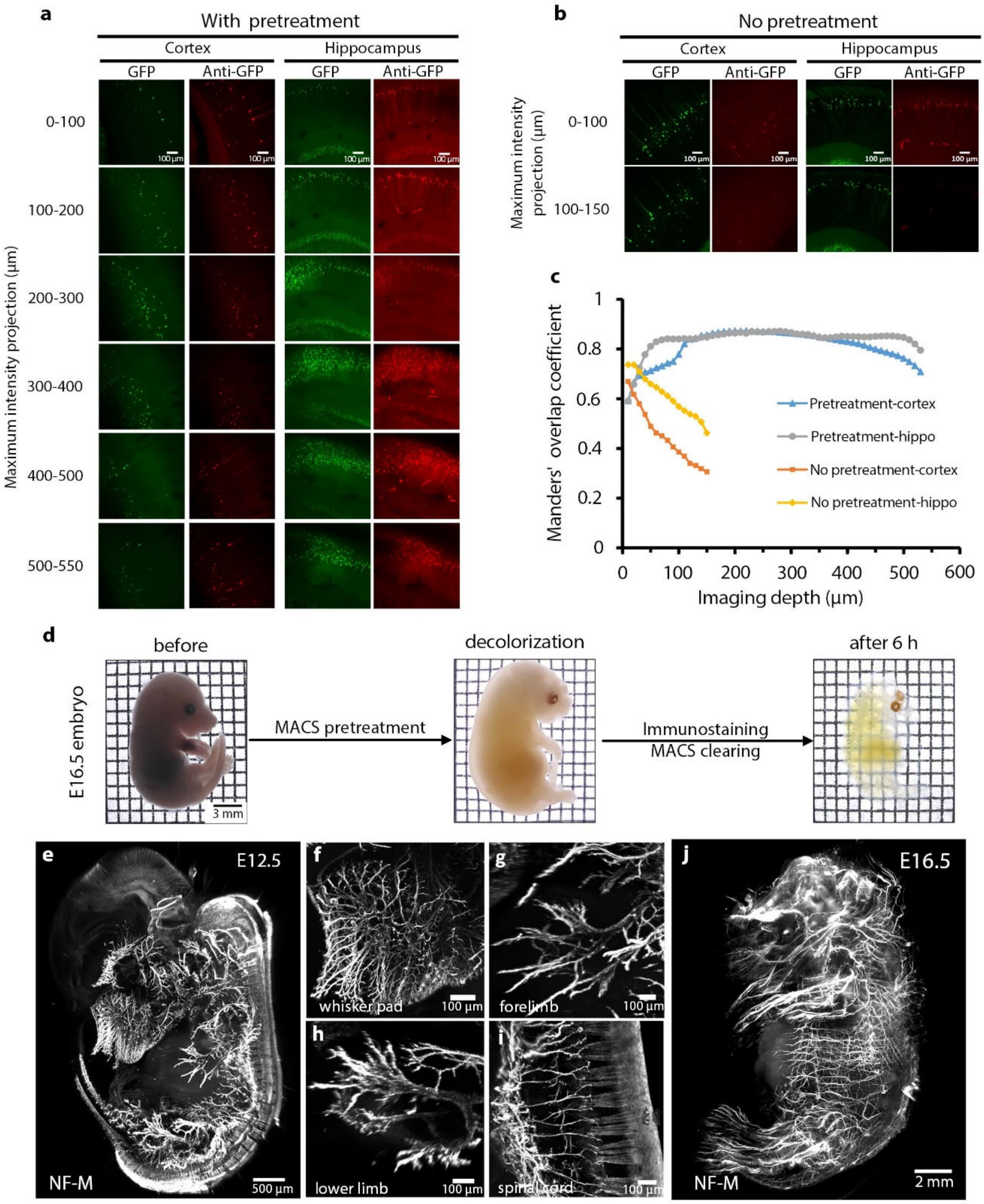
a
With pretreatment
Cortex
Hippocampus
GFP
Anti-GFP
GFP
Anti-GFP
Maximum intensity projection (μm)
0-100
100-200
200-300
300-400
400-500
500-550
100 μm
b
No pretreatment
Cortex
Hippocampus
GFP
Anti-GFP
GFP
Anti-GFP
Maximum intensity projection (μm)
0-100
100-150
c
Manders' overlap coefficient
Imaging depth (μm)
Pretreatment-cortex
Pretreatment-hippo
No pretreatment-cortex
No pretreatment-hippo
d
E16.5 embryo
before
MACS pretreatment
decolorization
Immunostaining
MACS clearing
after 6 h
3 mm
e
E12.5
NF-M
500 μm
f
whisker pad
100 μm
g
forelimb
100 μm
h
lower limb
100 μm
i
spinal cord
100 μm
j
E16.5
NF-M
2 mm

Figure S8. Whole-mount immunostaining of samples pre-treated by MXDA solution. (**a** to **c**) Promotion of antibody penetration of tissue treated by MXDA solution. Confocal images of MXDA-treated (**a**) and non-treated (**b**) samples stained by anti-GFP antibody. Maximum intensity projection images in indicated depth along the z axis were shown. Brightness of the images were adjusted by Fiji at minimum when visualized. (**c**) Manders' overlap coefficient was calculated by Fiji. (**d** to **j**) Whole-mount staining and imaging of intact embryos. (**d**) Work flow for immunostaining and clearing of whole embryos (e.g., E16.5). (**e**) Whole-mount immunostaining of E12.5 embryo labeled for anti-neurofilament (NF-M). (**f** to **i**) Details of the labeled neural structures at E12.5 in the whisker pad (**f**), forelimb (**g**), lower limb (**h**) and spinal cord (**i**). (**j**) 3D rendering of whole E16.5 embryo labeled for NF-M.

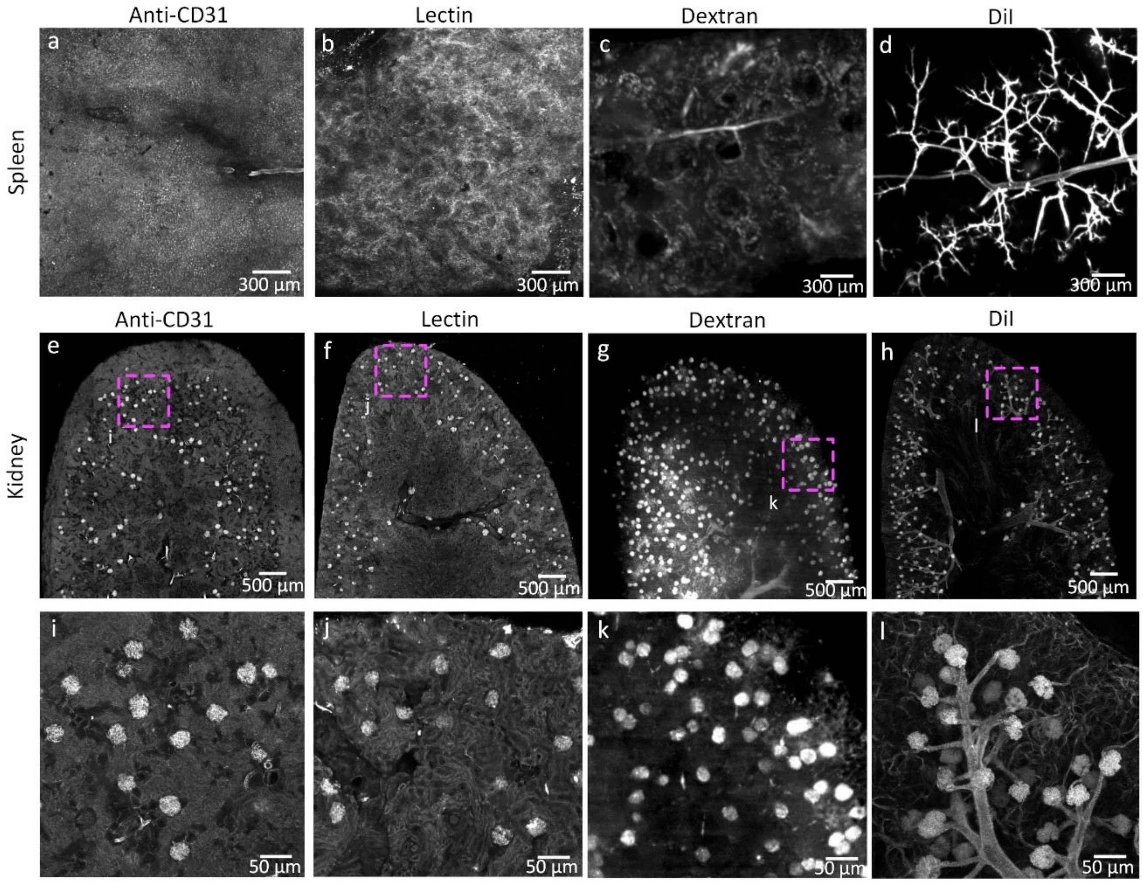

Figure S9. Comparison of different vasculature labeling methods on typical organs. (**a** to **d**) Vascular labeling of mouse spleens using anti-CD31 antibody (**a**), lectin (**b**) , dextran (**c**) and DiI (**d**), respectively. (**e** to **h**) Vascular labeling of mouse kidneys using anti-CD31 antibody (**e**), lectin (**f**), dextran (**g**) and DiI (**h**), respectively. (**i** to **l**) Magnified images of boxed regions in **e** to **h**, respectively. The glomeruli were well labeled by four labeling methods, but the surrounding vessel structures were only visible in DiI labeled kidneys.

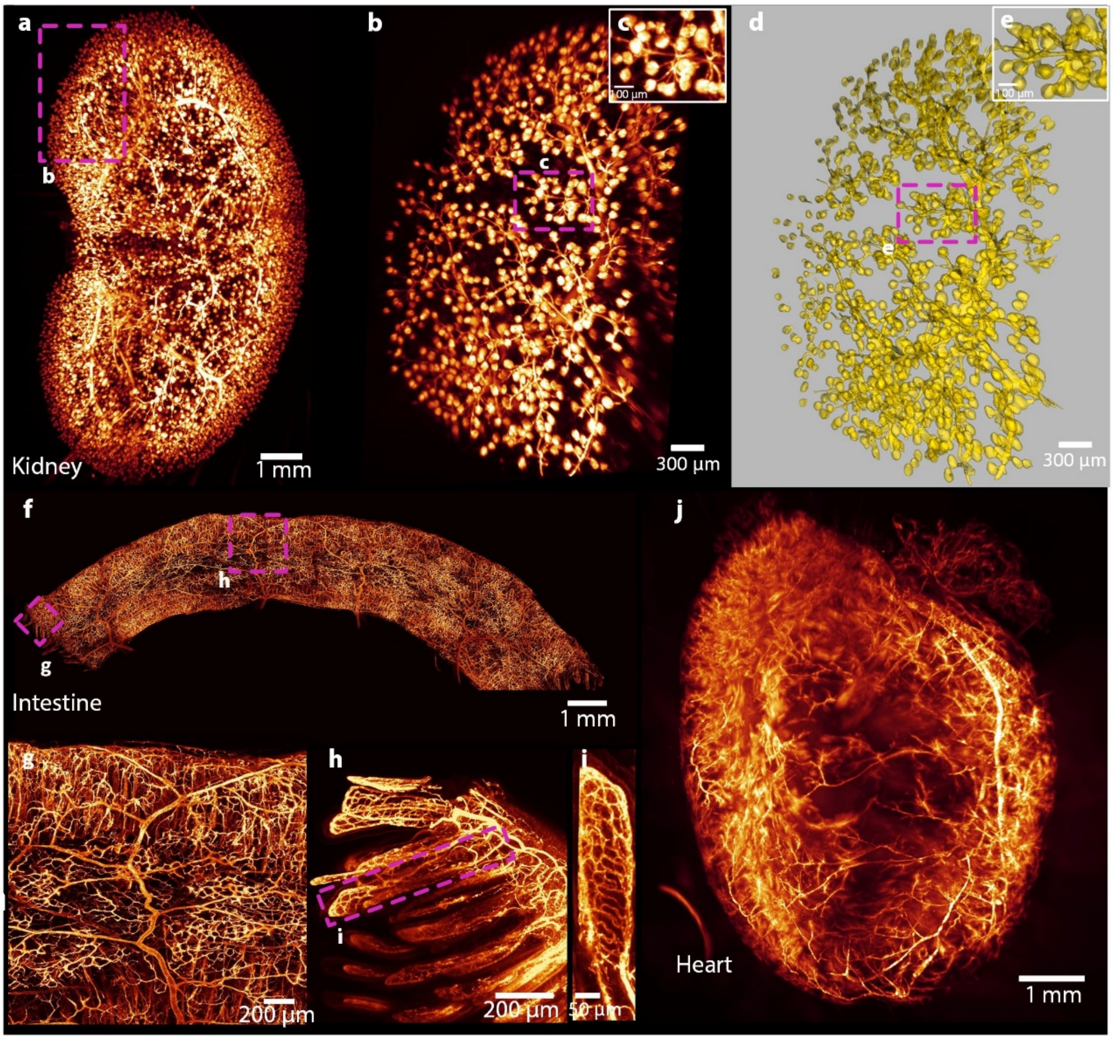

Figure S10. 3D visualization of the vascular networks of the mouse kidney, intestine and heart. (**a**) 3D reconstruction of the vascular network throughout the kidney. (**b**) Magnification of boxed region in **a**. The glomeruli and attached capillaries are well identified (**c**). (**d** to **e**) Volume rendering of structures shown in **b** and **c**. (**f**) 3D reconstruction of the intestine vasculature. (**g** to **h**) High-magnification images of the boxed regions in **f**, showing the vessel network (**g**) and villi (**h**) on the outer and inner surface of the intestine, respectively. (**i**) High-magnification images of the boxed region in **h**, showing the detailed structure of a single villus. (**j**) 3D rendering of the vasculature of an intact mouse heart.

Table S1. Changes in the pH of different agents after decolorization

Agents	50 vol% Quadrol	0.01 M NaOH	50 vol% MXDA
pH (before)	10.5	12.0	11.5
pH (after)	9.8	10.6	11.4

The pH values of the three solutions were measured before and after decolorization of embryos containing blood. The pH values of Quadrol and NaOH solutions revealed obvious decline, while the pH of MXDA solution remained stable.

Movie S1. LSFM imaging of MACS-cleared mouse brain (*Thy1*-GFP-M).

Movie S2. 3D rendering of neural networks of E14.5 immunolabeled embryo cleared by MACS.

Movie S3. 3D visualization of DiI labeled vasculature in mouse brain.

Movie S4. 3D visualization of DiI labeled vasculature in mouse spinal cord.

Movie S5. 3D rendering of DiI labeled vasculature in mouse spleen.

Movie S6. Reconstruction of the glomeruli in normal mouse kidney.

Movie S7. Reconstruction of the glomeruli in 4 W diabetic mouse kidney.
